# Genetic diversity and population structure of African village dogs based on microsatellite and immunity-related molecular markers

**DOI:** 10.1371/journal.pone.0199506

**Published:** 2018-06-25

**Authors:** Leona Vychodilova, Michaela Necesankova, Katerina Albrechtova, Jan Hlavac, David Modry, Eva Janova, Mirko Vyskocil, Andrei D. Mihalca, Lorna J. Kennedy, Petr Horin

**Affiliations:** 1 Department of Animal Genetics, University of Veterinary and Pharmaceutical Sciences, Brno, Czech Republic; 2 Department of Pathology and Parasitology, University of Veterinary and Pharmaceutical Sciences, Brno, Czech Republic; 3 Ceitec VFU, University of Veterinary and Pharmaceutical Sciences, Brno, Czech Republic; 4 Vétérinaires Sans Frontières Czech Republic, Brno, Czech Republic; 5 Biology Centre, Institute of Parasitology, Czech Academy of Sciences, České Budějovice, Czech Republic; 6 Department of Parasitology and Parasitic Diseases, Faculty of Veterinary Medicine, University of Agricultural Sciences and Veterinary Medicine Cluj-Napoca, Cluj-Napoca, Romania; 7 Centre for Integrated Genomic Medical Research, University of Manchester, Manchester, United Kingdom; National Cheng Kung University, TAIWAN

## Abstract

The village and street dogs represent a unique model of canine populations. In the absence of selective breeding and veterinary care, they are subject mostly to natural selection. Their analyses contribute to understanding general mechanisms governing the genetic diversity, evolution and adaptation. In this study, we analyzed the genetic diversity and population structure of African village dogs living in villages in three different geographical areas in Northern Kenya. Data obtained for neutral microsatellite molecular markers were compared with those computed for potentially non-neutral markers of candidate immunity-related genes. The neutral genetic diversity was similar to other comparable village dog populations studied so far. The overall genetic diversity in microsatellites was higher than the diversity of European pure breeds, but it was similar to the range of diversity observed in a group composed of many European breeds, indicating that the African population has maintained a large proportion of the genetic diversity of the canine species as a whole. Microsatellite marker diversity indicated that the entire population is subdivided into three genetically distinct, although closely related subpopulations. This genetical partitioning corresponded to their geographical separation and the observed gene flow well correlated with the communication patterns among the three localities. In contrast to neutral microsatellites, the genetic diversity in immunity-related candidate SNP markers was similar across all three subpopulations and to the European group. It seems that the genetic structure of this particular population of Kenyan village dogs is mostly determined by geographical and anthropogenic factors influencing the gene flow between various subpopulations rather than by biological factors, such as genetic contribution of original migrating populations and/or the pathogen-mediated selection. On the other hand, the study of oldest surviving dogs suggested a biological mechanism, i.e. a possible advantage of the overal heterozygosity marked by the the microsatellite loci analyzed.

## Introduction

The dog was the first domesticated mammal [1]. Molecular genetic data showed that the grey wolf (*Canis lupus*) was the ancestor of all current dog breeds and that the domestication process probably occurred in multiple locations in Asia [2–7]. The second phase of dog domestication was characterized by centuries of selective breeding resulting in more than 400 currently recognized breeds with extensive variation in size, shape, physiology, behavior as well as susceptibility to disease [8–13].

Following domestication, dogs quickly spread throughout Europe and to other continents. Specific populations referred as ‘‘village dogs” emerged worldwide, living as human commensals. These village dogs were not subject to the same degree of selective breeding and veterinary care as modern dog breeds. Progressively, they developed geographically characteristic genetic differentiation [14]. Modern village dogs seem to be complex mixtures of several non-native breeds and/or mixtures of both non-native breeds and indigenous village dogs [15–17].

Several types of markers can be used for assessing genetic diversity of specific populations. Microsatellites, mtDNA and biochemical markers were often used for this purpose [18]. Microsatellites and population characteristics based on these neutral markers characterize the general level of genetic diversity, population structure and evolutionary relationships between populations and subpopulations [19–22]. Microsatellites were used for characterizing neutral genetic variation between and within dog populations [8, 23] as well as for African village dogs [15]. It seems that the village dog populations contribute significantly to the huge genetic diversity of domestic dogs. The sequencing of the dog genome [3] accelerated the identification of a large number of single nucleotide polymorphisms (SNPs) and the use of these markers for genome wide studies, a novel type of polymorphic molecular marker that can be used for population studies [4, 15, 24–27]. This allowed the creation of SNP arrays, which can be used to genotype each dog for 170,000 SNPs in one assay.

Individual genetic variation in microsatellite loci does not necessarily correlate with that at expressed, especially non-neutral loci [28, 29]. The variability at microsatellite loci is affected by the numbers of alleles, and at the population level, it may change more rapidly in microsatellites than in protein coding regions. Different markers thus provide different information about levels of diversity and levels of selection in the populations studied. Neutral markers, such as microsatellites, inform on a population´s genetic structure with little evidence of positive and/or negative selection, while analysis of potentially non-neutral loci may reveal further sources of population differentiation.

Applications of SNPs for population genetics require evaluation of the markers used for a specific population, as information obtained based on SNPs in neutral and non-neutral loci may differ [29]. In domestic dogs, SNPs located in specific genes were shown to be informative markers of important traits and diseases [30, 31, 32, 33]. Genetic diversity and domestication of dogs have also been investigated with SNPs [4, 33, 34].

Immune-related (IR) genes were shown to be strongly targeted by selection, most likely pathogen-driven [35]. Subject to different selection pressures, they represent a model example of non-neutral variation [36]. Among functionally important IR genes, the Major Histocompatibility Complex (MHC) genes represent a particularly suitable tool for investigating functionally important variation and natural selection [37–40]. In addition, polymorphisms in other IR genes, such as cytokine-coding genes have been shown to represent informative markers for population diversity studies [41–43].

The African village dog populations are likely to be more adapted to local environmental conditions than purebred dogs [44]. Due to the absence of veterinary care and selective breeding, they thus represent a particularly well suited model for studying effects of this adaptation on IR genes. So far, studies exploring the genetic diversity of the immune genes in dogs have focused almost exclusively on MHC genes (known as DLA in the dog) [45,46]. Recently, we have shown that some polymorhisms in candidate IR genes are associated with susceptibility to multiple infections [47]. Taking into consideration all limitations of association studies, such as the size of the groups analyzed and/or a possible bias in the selection of candidate genes, we can expect that some of these markers might have adaptive values.

Comparisons of genetic diversity in neutral markers with non-neutral immune-related markers is one of the approaches to study the effects of selection due to host and pathogen interactions in both animal and human populations [48].

This study was based on the following hypotheses: i) The overall genetic diversity of the Kenyan village dogs is higher compared to European dogs due to absence of selective breeding; ii) The genetic substructure of the population is influenced by the pathogen pressure; iii) Differences in genetic diversity between neutral and potentially non-neutral (immune-response related) markers associated with infection in our previous study of this population may be interpreted as effects of a selective pressure of these pathogens; iv) Complex selection pressures will cause differences in the genetic diversity between the original population and a group of the oldest surviving dogs.

Therefore, we assessed the genetic diversity and population structure of an African village dog population with neutral (microsatellite) and non-neutral (IR candidate gene) markers and made a comparison with other African and European dogs.

## Materials and methods

All samples analyzed in this study had been originally collected for other purposes and were shared for these analyses as peripheral blood aliquots. All samples were collected by a licensed veterinarian in compliance with all ethical and professional standards. The field work in Kenya was approved by the Marsabit District Veterinary Office. The blood samples of European dogs were originally collected for diagnostic purposes at the Small Animal Clinic for a project accepted by the Internal Grant Agency of the University of Veterinary and Pharmaceutical Sciences Brno (Project #153/2008/FVL, Bone and joint infections of dogs as a biomedical model).

### The Kenyan village dog population and the study design

**Fig 1:**
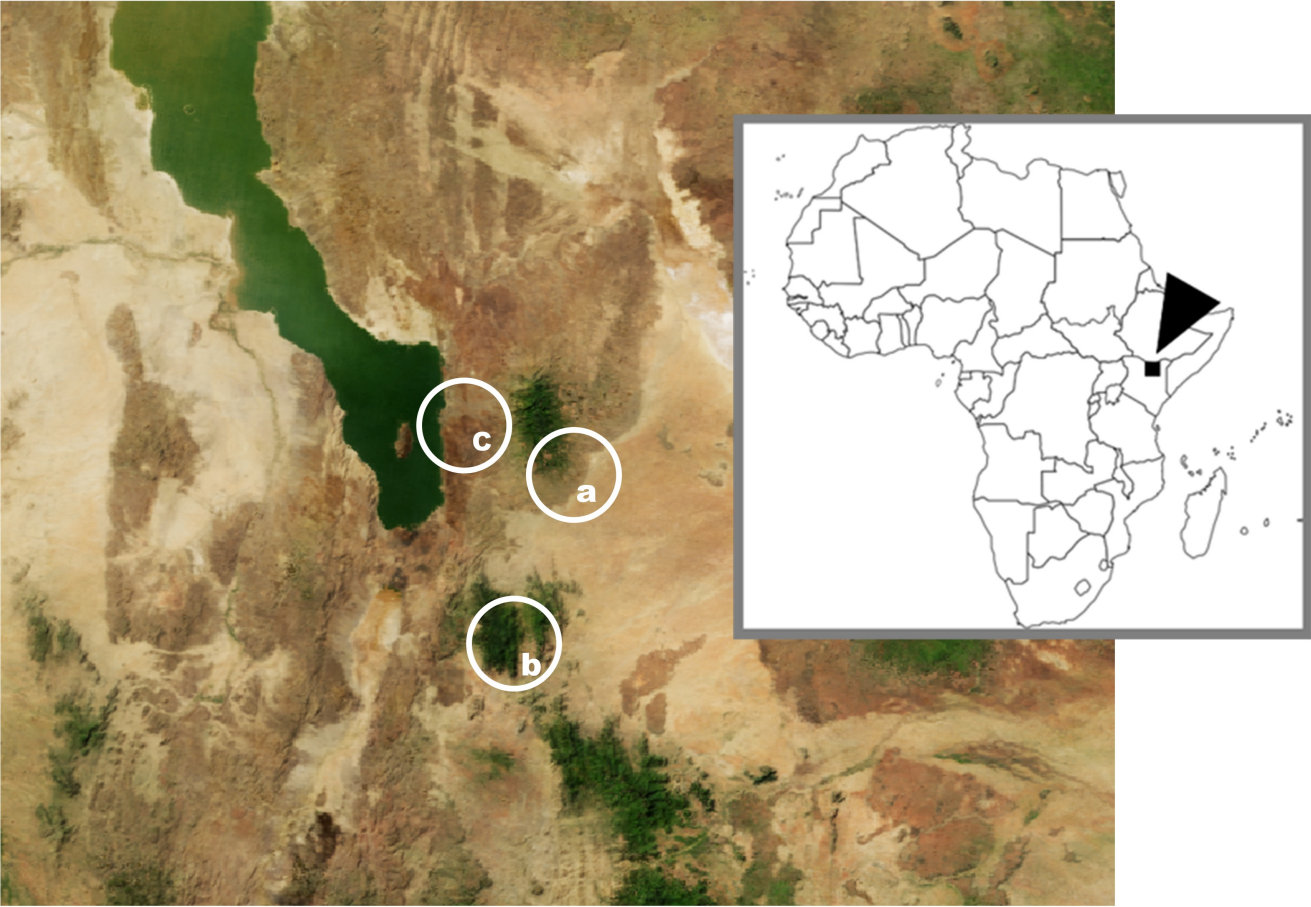
The region around the southern tip of the Lake Turkana in Northern Kenya. (A) Mt. Kulal. (B) Mt. Ngyiro. (C) Lake Turkana.

The African village dogs analyzed in this study are kept by semi-nomadic pastoralists of Samburu and Turkana tribes to guard the herds in three regions in Northern Kenya. Mt. Kulal (2335m) and Mt. Ngyiro (2752m) are two mountain ranges situated around the southern tip of Lake Turkana (410m) in the Rift Valley Province (Fig 1). Both mountain ranges are separated by vast areas of desert and semidesert habitat. The human population is concentrated in the more humid regions around both mountains (Samburu people) and close to the Lake Turkana shore around the local town Loiangalani (predominantly Turkana people). Due to ethnic reasons and separation by hostile desert/semidesert, there is limited connectivity between the three regions, including limited mobility of dogs. Although both the Samburu and the Turkana people belong to Nilotic tribes, their origin in the area is different. While the Turkana people seceded from the Karamojong people of West Uganda, part of the so called Karamojong cluster, Samburu are part of the Maa language group, representing the northern offshoot of Massai [49, 50]. Being pastoralists, both ethnics live closely with domestic dogs. In case of Turkana, their close human-dog association is even responsible for the highest incidence of hydatid disease in the world [51]. Regardless a permanent tension between both militant ethnics, the town of Loiyangalani (a center of our “Lake Turkana” sampling area) represents an important trade point, where Turkana and Samburu people use to meet.

**Fig 2:**
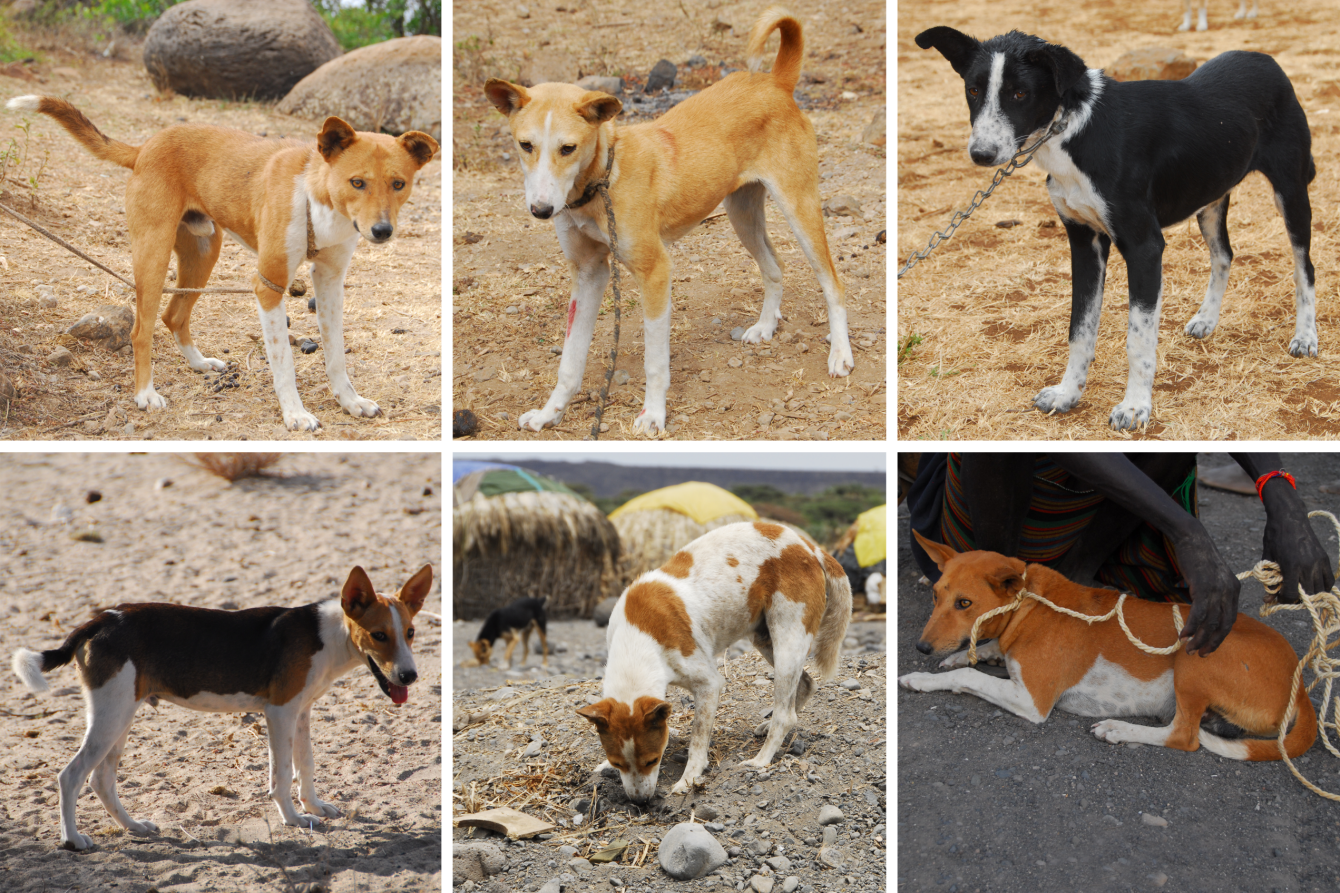
Phenotype of sampled dogs. Upper row—dogs of Samburu people from Mt. Kulal area, lower row–Turkana dogs from Lake Turkana (Loyiangalani) area.

Both the Turkana and Samburu pastoralists consider their dogs important helpers and guards of human settlements and livestock herds. Although the majority of these dogs are owned, having a name and belonging to particular families, the population lives in the villages with no veterinary care and no selective breeding. Most of dogs are short haired, of medium body size with various coat colors (Fig 2). The population is characterized by a high proportion of adolescent and young adult dogs; males are preferred to females by the owners. Infections, injuries and to some extent predation by large wild carnivores (leopards, hyenas) are the major reasons of mortality and the fast population turnover.

The genetic diversity of the three subpopulations of dogs living in 11 different villages—Mt. Kulal (Samburu people, 50 dogs, six villages), Mt. Ngyiro (Samburu people, 50 dogs, two villages) and Lake Turkana/Loiyangalani (predominant Turkana people, 50 dogs, three villages) was studied. In addition, the genetic diversity of a group of the oldest dogs identified in Mt. Kulal by the end of the study, was assessed using the same panel of markers. Dogs older than three years (“survivors”, n = 21) were available for this purpose. The age of the dogs was always indicated by the owner at the time of vaccination/blood collection and it was checked whether corresponded with the overall status of the particular dog and its dentition. These dogs represented 13.13% of the entire population. Comparisons of genetic diversity in different markers between this group and the original Mt. Kulal population were made.

The blood samples were obtained during the Mt. Kulal Rabies Control project, a long term anti-rabies vaccination programme [52].

### European dogs

Sixty-eight unrelated dogs—patients of the Small animal clinic of the University of Veterinary and Pharmaceutical Sciences Brno, representing the wide range of variation of European dogs were studied. The panel was composed of dogs of 24 breeds (Briard, English Cocker Spaniel, American Cocker Spaniel, Dachshund, Dobermann, Poodle, Howavart, Pit Bull Terrier, Weimaraner, German Shorthaired Pointer, American Staffordshire Terrier, Bearded Collie, Italian Greyhound, German Shepherd Dog, Yorkshire Terrier, German Boxer, Beagle, Belgian Shepherd Dog Malinois, German Spaniel, Parson/Jack Russel Terrier, Golden Retriever, Rottweiler, Whippet, Wolfhound) and six mongrels.

### Markers

#### Microsatellite genotyping

All dogs studied were typed on a panel of 27 microsatellite loci in two multiplex PCR protocols. Two commercial kits including loci recommended by the International Society for Animal Genetics and by the American Kennel Club for parentage testing and individual identification were used for this purpose. Genotyping was performed by the laboratory for parentage testing of the Institute of Animal Morphology, Physiology and Genetics at Mendel University in Brno, Czech Republic. The laboratory has participated to the comparison tests of the International Society of Animal Genetics (https://www.isag.us/comptest.asp) and used appropriate internal standards. The two sets comprised markers FHC2010, FHC2054, FHC2079, PEZ1, PEZ3, PEZ5, PEZ6, PEZ8, PEZ12, PEZ20 (Applied Biosystems, Foster City, CA, USA) and AHTk211, CXX279, INU055, REN169O18, REN54P11, AHT137, AHTh260, AHTk253, INRA21, REN169D01, AHT121, AHTh171, REN162C04, REN247M23, FHC2848, INU005, INU030 (Finnzymes diagnostic, Finland), respectively. The analysis was performed on an ABI PRISM 310 automated sequencer following manufacturers´ instructions.

#### SNP genotyping

A panel of 16 single nucleotide polymorphism (SNP) markers within nine immunity related candidate genes (*NOS3*, *IL6*, *TLR1*, *TLR2*, *TLR4*, *TLR7*, *TLR9*, *LY96*, *MYD88*) developed in our laboratory (Table 1) was used for genotyping. The genes were selected with respect to their potential importance in immunity against the diseases occurring in the population. Four infectious pathogens were diagnosed in the population under study: C*anine distemper virus*, *Hepatozoon canis*, *Microfilariae*, *Neospora caninum*. Detailed information on DNA sample collection and SNP genotyping is provided in [47].

**Table 1:** Immunity-related SNP markers.

Candidate gene	Gene/SNP symbol	Marker position in genome CanFam3.1	Type of SNP
Nitric oxide synthase	*NOS3/a*	Cfa16:g.15070013 A>T	intronic
	*NOS3/b *	Cfa16:g.15070184 A>C	intronic
Interleukin 6	*IL6/a*	Cfa14.g.36475649 A>G	intronic
	*IL6/b *	Cfa14:g. 36475658 C>T	intronic
Toll- like receptor 1	*TLR1*	Cfa3:g. 73543916 C>T	exonic (S)
Toll- like receptor 2	*TLR2*	Cfa15:g.51463020 C>A	exonic (NS)
Toll- like receptor 4	*TLR4/a*	Cfa11:g. 71365120A>G	exonic (NS)
	*TLR4/b *	Cfa11:g. 71366496G>C	exonic (S)
Toll- like receptor 7	*TLR7*	Cfa X:g.9356198C>A	exonic (S)
Toll- like receptor 9	*TLR9/a*	Cfa20:g.37545601 A>G	exonic (S)
	*TLR9/b*	Cfa20:g.37546031 A>G	exonic (NS)
Lymphocyte antigen 96	*LY96/a*	Cfa29:g. 22493202 C>T	intron/5´UTR
	*LY96/b*	Cfa29:g. 22493379A>G	intron/5´UTR
Myeloid differentiation primary response gene	*MYD88/a*	Cfa23:g.7901691 C>T	exonic (NS)
	*MYD88/d*	Cfa23:g.7902223 G>A	Intronic
	*MYD88/e*	Cfa23:g.7902449 C>T	Intronic
	*MYD88/b*	Cfa23:g. 7903927 C>G	intronic
	*MYD88/c*	Cfa23:g. 7904352 C>T	3´UTR
	*MYD88/f*	Cfa23:g. 7903760 T>A	Intronic
	*MYD88/g*	Cfa23:g. 7904004 G>C	Intronic
	*MYD88/h*	Cfa23:g. 7904166C>A	Exonic

S- synonymous substitution, NS- non synonymous substitution

#### MHC genotyping

135 Kenyan village dogs were genotyped in three DLA class II genes (*DLA-DQA*, *DRB*, *DQB*) as described in [53, 54, 55]. Three-locus haplotypes were first determined for individuals homozygous for each locus. In heterozygotes, most of the haplotypes were inferred based on the haplotypes observed in homozygous state. If haplotypes were only observed in heterozygotes, they were identified by subtraction of known haplotypes from heterozygous individuals [54]. Taking into consideration limitated availability of MHC genotyping, information from published studies on European dogs was used for comparison in this type of marker.

### Data analysis

Observed and expected heterozygosities, allelic richness, Hardy-Weinberg equilibrium (HWE), population pairwise F_ST_ values and Tajima´s test of selective neutrality were computed using Arlequin v3.11 [http://cmpg.unibe.ch/software/arlequin3] [56]. The Fisher 's exact test was used to compare allele frequencies between the European, Survivor, and African dogs. The extent of gene flow between populations (Nm) was calculated from the F_ST_ values using the formula Nm = (1/FST– 1)/4 [57]. Differences of mean heterozygosities between populations were analyzed by a standard Mann-Whitney-Wilcoxon test using the Statistica 6.0 software package [58]. The PHYLIP v3.6.9.5 software was used to compute Reynolds genetic distances [59].

The Bayesian clustering algorithm implemented in the STRUCTURE v2.3.4. software [60] was used to assess the number of ancestral populations underlying the dogs analyzed. Both the microsatellites and SNPs datasets were used for the model-based Bayesian clustering method. The admixture model with correlated allele frequencies was adopted for this purpose. Each parameter set was analyzed with five replicates for K = 2 to K = 7 and all runs were performed with 10000 burnin period and 50000 MCMC repeats after burnin. The optimum K value was assessed based on delta K values from Structure Harvester [61]. We followed the the software manual, which recommends to select the lowest value of K capturing the maximum degree of structure detected in the data. All K values presented here were selected in this way. A close match among iterated runs was found by CLUMPP software v. 1.1.2 [62]. CLUMPP results were used to generate bar graphs using DISTRUCT program. v. 1.1. [63]. Principal coordinates analysis (PCoA) via covariance matrix with data standardization was used (GENAIEx 6.5) [64] to partition the genetic variance among different subpopulations.

## Results

### Comparisons between Kenyan and European dogs

#### Microsatellite loci

For the population structure analysis, the value K = 3 for the microsatellites was estimated as the most informative. While the group of European dogs was represented by a single major cluster (S1 Fig), the entire population of Kenyan village dogs was found to be composed of three genetic clusters. One of these clusters included also the European group. The contribution of this group was low (S1 Fig).

Parameters of genetic diversity based on 27 microsatellites are shown in Tables 2, S1, S2 and S3 (Supporting information). The parameters of overall genetic diversity of Kenyan dogs was slightly higher compared to values observed in European domestic dogs. Kenyan dogs showed higher observed heterozygosities (Ho). In the Kenyan population, Ho was lower than He in the majority of microsatellite loci. The mean numbers of observed alleles per locus and effective alleles per locus were similar in both populations. Unique alleles were found in both populations, ranging from 17 (Kenyan dogs) to 30 (European dogs). More loci displayed departures from Hardy–Weinberg equilibrium (deficiency of heterozygous genotypes) in European dogs (21/27 = 77.7%) as compared to village dogs (13/27 = 48.1%). The genetic distance between European and Kenyan dogs expressed as an overall F_ST_ value was low (Table 3).

**Table 2:** Heterozygosities in Kenyan village, European and Survivor dogs.

Marker	Mean observed heterozygosity (Ho±SEM)		Mean expected heterozygosity (He ±SEM)		P values (Kenyan x European dogs)		Number of loci departed from HWE	
	**Kenyan**	**European**	**Kenyan**	**European**	**Ho**	**He**	**Kenyan**	**European**
	(n = 150)	(n = 68)	(n = 150)	(n = 68)			(n = 150)	(n = 68)
**Microsatellites**	0.69± 0.02	0.64± 0.02	0.77± 0.02	0.79± 0.01	0.003	NS	13	21
**SNPs**	0.31± 0.04	0.30± 0.03	0.36± 0.04	0.40± 0.03	NS	NS	1	7
	**Survivors**	**The original Mt. Kulal dogs**	**Survivors**	**The original Mt. Kulal dogs**	**P values** (Survivors x The original Mt. Kulal dogs)		**Survivors**	**The original Mt. Kulal dogs**
	(n = 21)	(n = 39)	(n = 21)	(n = 39)			(n = 21)	(n = 39)
**Microsatellites**	0.77± 0.02	0.68± 0.03	0.77± 0.02	0.77± 0.01	2x10^-4^	NS	5	8
**SNPs**	0.36± 0.06	0.28± 0.04	0.33± 0.04	0.32± 0.04	NS	NS	1	1

Ho heterozygosity observed, He heterozygosity expected, SEM—standard error of the mean, NS–non-significant

**Table 3:** Pairwise FST values and Nm values in Kenyan village and European dogs based on microsatellite, SNPs and MHC markers.

	FST			Nm
	SNPs	Msats	MHC	Msats
**Mt. Kulal- Mt. Ngyiro**	0.054*	0.035*	0.027*	5.94
**Mt. Kulal- Lake Turkana**	0.019*	0.017*	0.001^NS^	12.7
**Mt. Ngyiro- Lake Turkana**	0.024*	0.028*	0.027*	6.51
**European-Kenyan**	0.071**	0.049**	NC	4.87
**European- Mt. Kulal**	0.099**	0.046**	NC	5.17
**European-Mt.Ngyiro**	0.043**	0.057**	NC	4.16
**European-Lake Turkana**	0.070**	0.046**	NC	5.23

^NS^ Non-significant;

*P<0.05;

**P<0.01,

NC not calculated

#### Single nucleotide polymorphisms in candidate IR genes

The population structure analysis of IR gene SNP markers revealed that individual dogs represented mixed contributions from inferred ancestral populations (S2 Fig). The proportions assigned to each cluster at K = 3 are subtly assymetrical in the European dogs; they could be distinguished from Kenyan village dogs.

Parameters of genetic diversity in all immune SNP markers tested and comparisons between Kenyan and European dogs are summarized in Tables 2, S4, and S5. In both groups, all loci were polymorphic and bi-allelic. While all three possible genotypes in all loci analyzed were observed in European dogs, only two genotypes (one homozygous and the heterozygous genotype) were found in two loci (*IL6a*, *TLR2*) in African dogs. Significant differences (P<0.01) in allelic frequencies between European and African dogs were observed for loci *NOS3a*, *IL6b*, *TLR2*, *TLR4b*, *TLR7*, *TLR9a*,*b*, *LY96a*,*b*, and *MYD88a*. Observed and expected heterozygosities were similar in both groups. Seven SNP loci were out of HWE (deficiency in heterozygous genotypes) in European dogs compared to one locus (*TLR7*) in village dogs (Table 2). In addition, genetic distances between the two groups expressed as F_ST_ values computed for microsatellite and SNP loci showed bigger distance for SNP loci (Table 3). The gene flow between European and Kenyan dogs calculated based on the microsatellite loci was 4.87 migrants per generation (Table 3).

### Analysis of the Kenyan village dog population

#### Microsatellite loci

**Fig 3:**
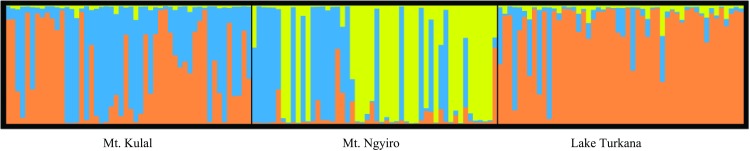
Estimation of the population substructure across microsatellite loci in the Kenyan village dogs (K = 3) using Structure software. A different pattern was identified for Mt. Ngyiro.

When the population substructure was assessed, Mt. Ngyiro dogs showed different pattern of clustering compared to Mt. Kulal and Lake Turkana (Fig 3). The cluster with the highest contribution to the Mt. Ngyiro subpopulation (0.529) showed the lowest contribution to the other two subpopulations (0.019 for Mt. Kulal and 0.039 for Lake Turkana).

**Fig 4:**
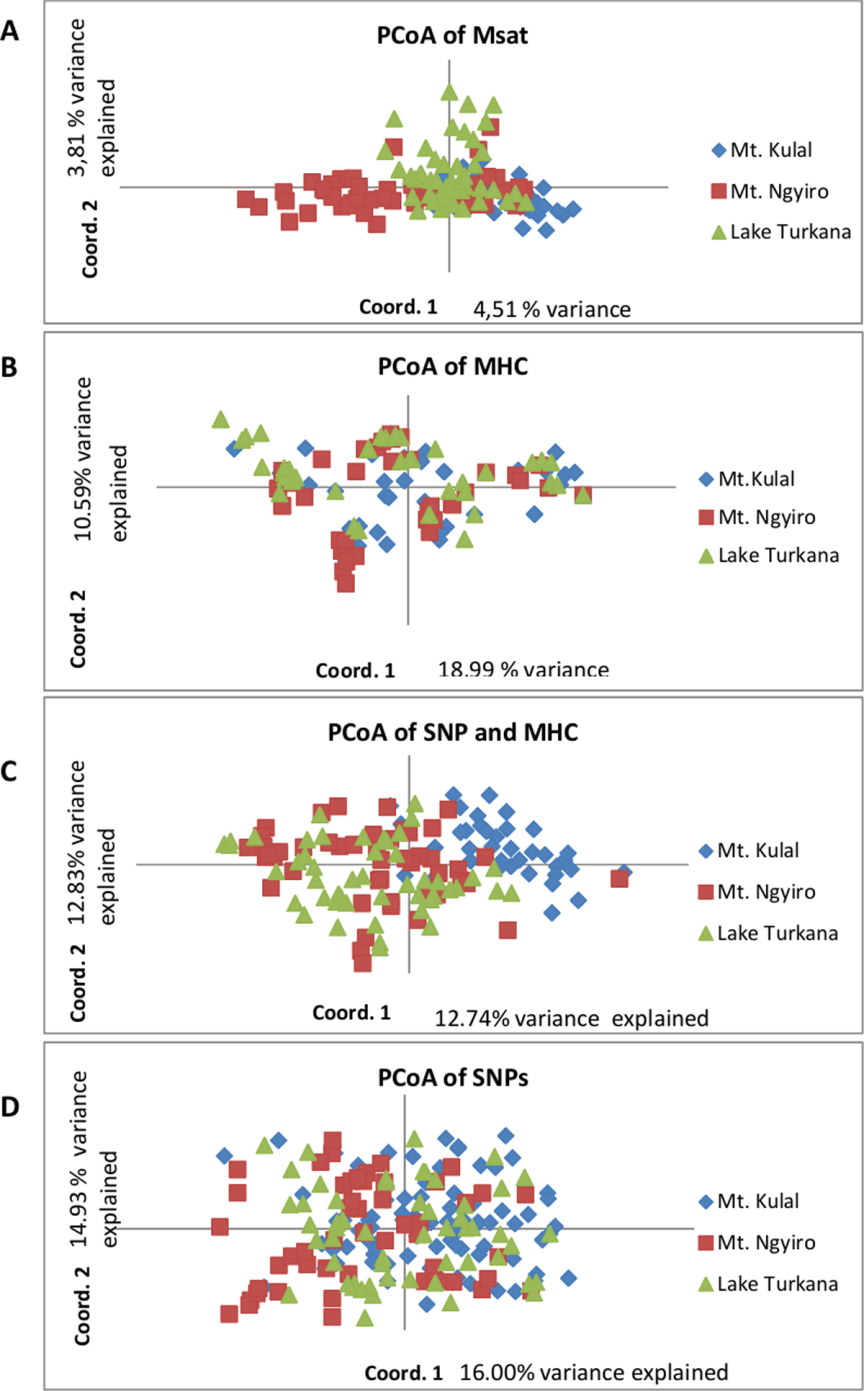
Principal coordinates analysis (PCoA) of the Kenyan village dogs. (A) PCoA with the 27 microsatellite loci (n = 150). (B) PCoA with the 3 MHC loci (n = 135). (C) PCoA with the 3 MHC and 16 SNP loci (n = 135). (D) PCoA with the 16 SNP loci (n = 150).

These results are supported by the principal coordinates analysis (PCoA, Fig 4A). The first axis shows that the distinction between Mt. Ngyiro and Mt. Kulal dogs explained 4.51% of the overall variance. The second axis shows that 3.81% of the overall variance separated Lake Turkana dogs.

The genetic diversity of the three Kenyan village dogs subpopulations identified by microsatellite analysis was further characterized and compared (Tables 4, S3, S6, S7 and S8). The Mt. Ngyiro subpopulation had significantly lower mean heterozygosity (both Ho and He) and mean number of alleles of microsatellites than Lake Turkana (P = 3.7x10^-7^) and Mt. Kulal (P = 0.009) dogs. A lower number of effective alleles per locus was found in Mt. Ngyiro when compared to Mt. Kulal dogs. Mt. Kulal showed significantly (P = 0.013) lower observed heterozygosity values than Lake Turkana, while values of expected heterozygosities were similar.

**Table 4:** Heterozygosities in three subpopulations of Kenyan village dogs.

	Mean heterozygosity observed (SEM)			Mean heterozygosity expected (SEM)			P values (Observed heterozygosity)		
Marker	Mt. Kulal	Mt.Ngyiro	Lake Turkana	Mt. Kulal	Mt.Ngyiro	Lake Turkana	1x2	1x3	2x3
	(n = 50)	(n = 50)	(n = 50)	(n = 50)	(n = 50)	(n = 50)	0.009	0.013	3.7x10^-7^
**Microsatellites**	0.69± 0.02	0.63± 0.02	0.74 ± 0.02	0.77± 0.01	0.73 ± 0.02	0.77 ± 0.01			
	(n = 84)	(n = 50)	(n = 50)	(n = 84)	(n = 50)	(n = 50)	NS	NS	NS
**SNPs**	0.30 ± 0.04	0.33 ± 0.04	0.31 ± 0.05	0.32 ± 0.04	0.38 ± 0.04	0.34 ± 0.05			
	(n = 41)	(n = 47)	(n = 47)	(n = 41)	(n = 47)	(n = 47)	NS	NS	NS
**MHC**	0.75± 0.08	0.87± 0.02	0.85± 0.08	0.79 ± 0.08	0.88± 0.03	0.82± 0.07			

1 Mt. Kulal, 2 Mt. Ngyiro, 3 Lake Turkana, SEM—standard error of the mean, NS—non-significant

#### MHC loci

For MHC markers, the population substructure analysis revealed three slightly unequally distributed clusters, where Mt. Ngyiro could be distinguished from Mt. Kulal and Lake Turkana (S3 Fig). This substructure was much less distinct when compared to the results based on microsatellite markers.

The numbers of alleles in individual MHC loci were 10 for *DQA*, 17 for *DQB* and 25 for *DRB1*. Seven new *DRB1* alleles and one new *DQB1* alleles were identified. All the new alleles were found on specific haplotypes, as shown in Table 5 apart from one of the *DRB1* alleles (094v). The highest values of mean expected (0.88± 0.03) and observed heterozygosities (0.87± 0.02) were found in Mt. Ngyiro dogs, with non significantly lower heterozygosities in both Lake Turkana and Mt. Kulal dogs. No departure from HWE was observed. The *DQA1* locus showed the lowest heterozygosity (Tables 4, S9 and S10).

**Table 5:** DLA three locus class II haplotypes in three Kenyan villages (%).

haplo ID	*DRB1*	*DQA1*	*DQB1*	# dogs	# homo-zygous dogs	Lake Turkana n = 47	Mt. Kulal n = 41	Mt. Ngyiro n = 47	Relative frequencies	Survivors n = 28
1	00101	00101	00201	21	1	0.14	0.04	0.05	TkN	0.09
2	00201	00901	00101	5		0.02	0.04	0	tK	0.0
3	00301	00101	00802	2		0.02	0	0	T	0.02
4	00401	00201	01501	16		0.09	0.09	0.01	TKn	0.05
5	00601	005011	00701	15	2	0.02	0	0.14	tN	0.0
6	00802	00301	**ug115**	12	1	0	0.02	0.11	kN	0.07
7	00901	00101	00802	24		0.10	0.08	0.10	TKN	0.10
8	00901	00101	008011	16		0.05	0.12	0.01	TKn	0.05
9	00901	00201	01305	5		0.02	0	0.03	tn	0.02
10	01101	00201	01302	2		0	0	0.02	n	0.0
11	01201	00401	nt	2	1	0	0.02	0	k	0.0
12	01301	00101	00201	12	1	0	0.02	0.11	kN	0.04
13	01501	00601	02301	14		0.10	0.01	0.04	TkN	0.07
14	01501	00601	03101	11		0.09	0.01	0.02	Tkn	0.02
15	01501	00601	05401	9		0.02	0.02	0.06	tkN	0.05
16	01501	00601	05701	12		0.06	0.01	0.05	TkN	0.0
17	01801	00101	00802	8		0.01	0.09	0	tK	0.05
18	02001	00401	01303	16		0.03	0.01	0.13	tkN	0.07
19	04801	00402	02301	3		0	0.04	0	K	0.02
20	**10801**	00101	008012	32	4	0.08	0.26	0.04	TKN	0.14
21	**092v**	00601	00201	13	1	0.04	0.10	0.01	TKn	0.04
22	**108v**	00101	008012	5		0.04	0.01	0	Tk	0.02
23	**ph211**	00402	02301	4		0	0	0.04	N	0.04
24	**ph271**	00601	05701	5		0.02	0.01	0.02	tkn	0.02
25	**ph486**	00301	03801	4		0.04	0	0	T	0.0
26	07401	005011	00701	1		0	0	0.01	Single dog	0.0
27	**098v**	00402	02301	1		0.01	0	0	Single dog	0.02
				270	11	1	1	1		1

Alleles in bold are new alleles found in this study; official names are being identified. The “Relative frequencies” column indicates the relative haplotype frequencies, using lower (<4%) and upper (= 4% or more) case letters for each region: T = Lake Turkana, K = Mt. Kulal, N = Mt. Ngyiro. Frequencies (%) highlighted in red/blue indicate which region has the highest/lowest frequency of that haplotype, respectively.

In the entire group of 135 dogs, 27 different DLA three locus class II haplotypes were identified, found in between two and 32 different dogs; two were only found in single dogs. There were 11 dogs that were homozygous at all three loci.

**Fig 5:**
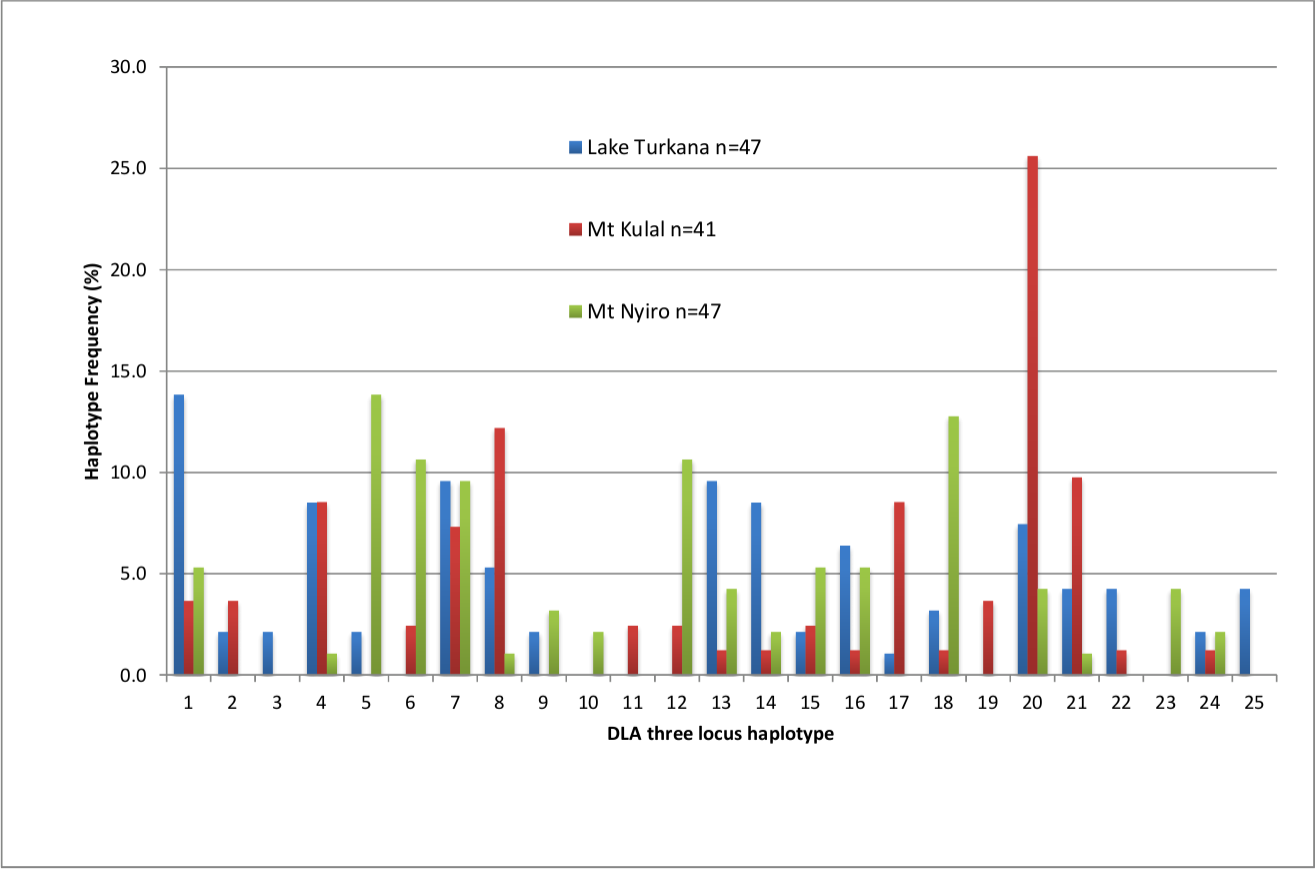
DLA three locus haplotype frequency profiles for the different subpopulations of the Kenyan dogs.

Twelve haplotypes were found in all three villages, while each village had two unique haplotypes. Each village also lacked two or three haplotypes that were present in the other two. However, each haplotype generally had a much higher frequency in one village compared to the other two (see frequencies in red in Table 5). Fig 5 shows the haplotype profiles for each region. Haplotype 1 was most frequent in Lake Turkana, haplotype 20 in Mt Kulal and haplotypes 5 and 18 in Mt Ngyiro.

#### Single nucleotide polymorphisms in candidate IR genes

**Fig 6:**
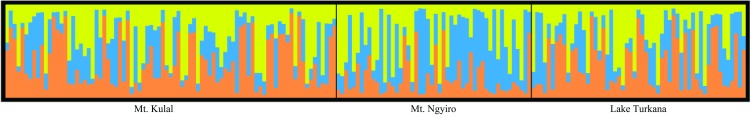
Estimation of the population substructure across SNP loci in the Kenyan village dogs (K = 3) using structure software. No substructure was identified.

No evidence for a specific substructure was found in the Kenyan dogs using the Structure software; all clusters contributed similarly to the three subpopulations studied (Fig 6). Since no clear structure was observed for any K values, no informative K could be determined. One graph was selected as a typical example (Fig 6). This finding was also supported by the PCoA analysis, where no separation of the three subpopulations was observed (Fig 4D).

The genetic diversity observed in expressed SNP marker loci was comparable in all three subpopulations (Tables 4, S4 and S11). Significant differences in allelic frequencies among the three subpopulations were observed for SNP markers *NOS3a*, *TLR4b*, *TLR9* (P<0.05), and for *IL6b*, *TLR1*, *LY96a* (P<0.01).

#### Comparisons among subpopulations

The F_ST_ pairwise comparisons among the three subpopulations for different types of markers are in Table 3. Taking into consideration that higher F_ST_ values indicate more isolation and differenatiation of the populations compared, the interpretation of the data is that most of the genetic variation in Kenyan dogs existed within the population, with rather small differences among the three subpopulations. In all types of loci, the genetic distances between Mt. Kulal and Lake Turkana dogs were smaller than distances between either of them and Mt. Ngyiro dogs. The gene flow rate estimates are in Table 3. The highest number of migrants per generation was estimated between Mt. Kulal and Lake Turkana (Nm = 12.70), the lowest values were observed for Mt Kulal and Mt. Ngyiro (Nm = 5.94).

Taken together, a clear substructure of the Kenyan village dog population was observed for microsatellite loci. No substructure was identified for candidate IR gene SNPs. A trend to sub-structuring was observed for MHC loci using Structure. No subpopulation differentiation was detected by the PCoA analysis of SNPs or MHC (Fig 4B, 4C, and 4D). The Reynolds genetic distances showed a significant correlation (r_S_ = 0.943, P = 0.005) only between MHC and microsatellite loci (S12 Table).

#### Genetic diversity of dogs older than 3 years (“survivor” study)

Parameters of genetic diversity in the group of oldest dogs and their comparison with the original Mt. Kulal population are shown in Table 2. The PCoA did not identify the group of survivors as a discrete subpopulation of the original Mt. Kulal population in any type of markers (S4 and S5 Figs). However, their genetic variability differed in microsatellites but not in the SNP loci analyzed. Survivor dogs showed significantly (P = 2x10^-4^) higher values of mean observed heterozygosity of microsatellites. The overall mean heterozygosity of IR gene SNP loci was also higher in the surviving dogs; however, this difference was not significant. Allelic variants of the genes *MYD88* and *LY96* associated with a lower risk of infection [47] were more frequent in this group (Table 6).

**Table 6:** Frequencies of diasease associated alleles [47] in survivor dogs and the original Mt. Kulal population.

Disease	Associated SNP	Allele	S-susceptibility/ R-resistance	Corrected P value	Frequency of allele		P values
					Survivors (n = 21)	Original Kulal dogs (n = 39)	
Canine distemper virus	*MYD88/a*	T	R	0.001	0.43	0.51	NS
microfilariaemia	*MYD88/a*	C	R	0.012	0.57	0.49	NS
Canine distemper virus	*MYD88/b*	G	R	0.025	0.52	0.51	NS
microfilariaemia	*MYD88/b*	C	R	0.019	0.48	0.49	NS
hepatozoonosis	***MYD88/c***	C	R	0.040	**0.93**	**0.77**	**0.023**
microfilariaemia	***MYD88/c***	T	S	0.003	**0.07**	**0.23**	**0.023**
neosporosis	***LY96/a***	T	S	0.012	**0.10**	**0.25**	**0.032**
microfilariaemia	***LY96/b***	A	R	0.019	**0.81**	**0.58**	**2x10**^**-4**^
neosporosis	*TLR1*	T	R	0.014	0.14	0.25	NS
neosporosis	*TLR4/a*	G	S	0.030	0.55	0.57	NS
hepatozoonosis	*TLR4/a*	G	R	0.045	0.55	0.57	NS
microfilariaemia	*NOS3b*	C	R	0.010	0.24	0.27	NS

NS—non-significant

## Discussion

The village and street dogs represent a unique model of canine populations. Although they always represent small and location specific samples, their analyses contribute to understanding general mechanisms governing their genetic diversity, evolution and adaptation [15, 65]. Besides studies on randomly selected village dog groups [65], a complex analysis of different populations from geographically separated regions and of their genetic structure is available [15]. The advantage of this study was our access to a population residing in the same area but belonging to different ethnics living in different regions, reflecting their complex geographical and socio-cultural relationships and interactions.

A comparison with a study where the same microsatellite marker set was used [20] showed that the variability of our dog population was comparable to other village dog populations. Studies using different microsatellite marker panels showed similar heterozygosities of various village dog populations (S13 Table). Slightly lower heterozygosities were found in village dogs from Egypt, Uganda and Namibia [15], similar values were reported for Bali street dogs [65]. Slightly higher values of heterozygosities were found in village dogs from Taiwan, Iran, Cook Island or Phillipines [20]. Like in other studies, our data showed an admixture of non-native breeds. It seems that the admixture of modern breeds was less common in Mt. Ngyiro. The lower gene flow to Mt. Ngyiro could reflect a bigger distance of this district from more popular locations like Lake Turkana and Mt. Kulal regions. Most village dogs had a proportion of less than 5% of the European cluster. These results support the idea that the ancestors of African village dogs brought to Kenya from Sudan during Nilotic migrations were influenced by modern breeds of dogs brought in by European settlers in the 17th century [66]. The finding a grey wolf MHC haplotype DLA-DRB1*00601/DQA1*005011/DQB1*00701 and DLA-DRB1*00901/DQA1*00101/ DQB1*008011 are in agreement with this assumption. However, the existence of dogs with high (>40%) contribution of the European cluster suggests recent admixtures, probably as a result of the previous colonial history and activities of several missions, operated in the area by European and American churches. These values are within the range reported by [15].

The European dogs studied here were selected with the primary purpose to cover the range of variation observed in Europe, and as such they do not represent a real genetic population. Therefore, in contrast to the Kenyan population, we did not analyze the detailed structure of the European group but we limited ourselves to its differentiation from African dogs. As mentioned above, the regular procedure in analyzing population structure is to select the lowest K value capturing the maximum degree of structure [67]. For our entire dataset, the optimum value was K3, which can be interpreted as the existence of three sub-populations where European dogs formed a cluster clearly different from the African village dogs. The parameters of overall genetic diversity, as assessed by microsatellite markers, were similar for both African and European groups. It means that the range (but not the structure) of genetic variation in this particular African village dog population is comparable to the range of variation observed in a group composed of many European breeds and mongrels. As most of genetic variation in dogs exists among breeds [25], the African population seems to have maintained a significant proportion of the existing genetic diversity of the canine species. The unique alleles of microsatellite markers document the extent of its specificity. In the context of the history of this population where admixture of different breeds occurred repeatedly, it shows that various polymorphisms distributed amongst different breeds in Europe have been successfully used by this population under significant selective pressure.

When compared with pure breed studies [8, 20, 68], the heterozygosity of African village dogs was usually higher with some exceptions (S14 Table) probably because heterozygosities are less sensitive parameters of genetic diversity than the number of alleles per locus [69]. The higher numbers of microsatellite loci that deviated from the HWE in European dogs can be explained by effects of selective breeding. The lower values of observed heterozygosity compared to expected heterozygosity observed in the Kenyan dog group can be explained by inbreeding and/or by genetic drift due to a partial isolation of the three subpopulations. Taking into account their expected frequencies, it does not seem likely that null alleles would significantly contribute to the excess of homozygotes observed. The three subpopulations differed significantly (P<0.05 and/or P<0.01) in their microsatellite heterozygosities, which underlines their specific genetic features.

African village dogs were shown to have a complex population structure, due to effects of geography, gene flow barriers, and the presence of non-native dogs [15]. The results of our population structure analysis and the F_ST_ values computed for microsatellite loci have shown that the population studied here is also clearly structured in three genetically similar but discrete subpopulations. In this sense, we may expect that factors determining its structure are similar to other African population, although their relative contribution is probably locally specific. A complex of biological factors, including inbreeding, genetic drift and/or various types of selection pressure as well as geographical, ethnic and social factors representing different gene flow barriers may contribute to the final overall structure.

Domesticated dogs had probably followed waves of Nilotic migrants to the south. The Samburu pastoralists as part of Maa people came to the area of today’s Kenya during the second half of the first millennium from southern Sudan, while origins of Turkana can be traced into Karamojong people of western Uganda and their migration eastwards in 17th century [49, 50]. Although originating from different migration waves, dogs from Mt. Kulal and Lake Turkana were closer each to the other than to the Mt. Ngyiro group, despite a different ethnicity of their owners. A high gene flow observed between them can be explained by a more active communication between people from these two locations in Loyiangalani, a center of trade, healthcare and offices of the local authorities. On the contrary, there is very little, if any, communication between two ethnically related mountain localities separated by the Chalbi desert. It thus seems that the genetic structure of Kenyan village dogs population corresponds to, and can be explained by gene flow predominantly determined by the ways of communication between human subpopulations inhabiting the area. This communication is influenced by geographical and social factors rather than by ethnicity. Besides possible effects of pathogen-driven selection discussed below, there are also further possible biological explanations for the structure observed, such as inbreeding, genetic drift or predation. Although the geographical and anthropogenic factors seem to fit the genetic data, we cannot exclude a contribution of e.g. genetic drift or inbreeding, especially with regards to the fact that in this population, observed heterozygosities were lower than expected. Taken together, the analysis of genetic diversity in potentially neutral markers showed that the Kenyan village dog population is similar to other African village dogs in terms of its genetic diversity, population structure and factors causing its diversification.

Although variation at neutral loci can give insights into genetic diversity within populations and genetic differentiation among populations, they provide only limited information on adaptive evolution [70]. The evolutionary dynamics of functional genes may be different and their patterns of genetic diversity can vary due to interplay between genetic drift and natural selection [71]. Analyses of these differences represent one of approaches of detecting effects of selection [48, 72]. Genome-wide SNPs scans have been successfully used for studying population structure and diversity of various mammalian species. However, these SNPs behaved as neutral markers [13, 15, 73]. Immune response genes, which are of direct adaptive value [74], are good markers for studying adaptive genetic variation of specific populations [75].

MHC genes are prime candidates for such studies. A comparison of three MHC genes (same like in this study) and a set of 17 neutral microsatellites was succesfully used for studying assessing effects of balancing selection in wolves by Niskanen et al. [76]. Here, we observed that the genomic MHC diversity of Kenyan dogs was higher than in the dog breeds studied so far [53,77,78]. When compared to the Bali street dogs [79], the numbers of alleles in Kenyan dogs were somewhat lower than in Bali dogs for all three loci analyzed, while their heterozygosities were comparable. Our group of 135 dogs had 27 different DLA three locus class II haplotypes. The numbers of haplotypes found in purebred dogs varied between two and twelve, so for these loci, Kenyan village dogs are more diverse than any domestic breed. Dogs homozygous at all three loci added support for the listed haplotypes. It is clear from Table 5 and Fig 5 that although most haplotypes are found in more than one village, each village has fairly closed dog communities, with a different haplotype being most frequent in each group.

As no data on non-MHC IR gene SNPs in African or other village dog populations are available, we only could make comparisons with whole genome SNPs and/or between non-MHC IR gene SNPs in African and European dogs. The genetic variation of 300 whole genome SNPs in African village dogs reported by [15] is slightly higher than values calculated with our markers. As it is not possible to make a direct comparison between genetic diversity in candidate non-MHC IR gene SNP markers and diversity of anonymous whole genome SNPs, we only can conclude that the overall genome SNP diversity does not seem to differ significantly from the diversity of the non-MHC IR loci studied. The parameters of genetic diversity in candidate non-MHC IR genes were similar for the African village and European dogs. Interestingly, in two loci (*IL6*, *TLR2*), one genotype was not found in the African village dogs, while in the European group, all three possible genotypes were observed. As both loci are in HWE, this is due probably to low frequencies of the minor alleles. However, the reason for the low frequencies remains unknown.

A comparison between the three groups of markers shows that the genetic structure defined by neutral microsatellites could not be observed for potentially non-neutral non-MHC IR loci. The differences between IR gene SNPs and microsatellite loci are probably due mostly to differences in their numbers of alleles. On the other hand, the patterns on multi-allelic MHC genetic diversity were closer to multi-allelic microsatellites rather than to bi-allelic SNP loci (S12 Table, S3 Fig).

Schierup et al. [80] showed that pathogen pressure tends to homogenize allele frequencies and reduce population differentiation based on MHC loci, and that in subdivided populations, balancing selection leads to lower expected F_ST_ values even for neutral sites linked to the selected locus. Therefore, weak effects of pathogen-mediated selection cannot be excluded, but the Tajima´s test of selective neutrality failed to detect them (S15 Table). These findings support the view that it is difficult to observe effects of pathogen-mediated selection on MHC loci in natural populations [48].

The village dogs are considered to live in conditions comparable to those during early stages of dog domestication. Infectious diseases are considered to be the main cause of morbidity and mortality in village dogs [81]. In this study, only few dogs older than three years could be found. Devastating infections, like distemper and rabies, but also to chronic infectious diseases reducing fitness are likely to be the major causes. Predators and the absence of specific care can also contribute to the rapid turnover. Although the group of oldest dogs does not represent a discrete subpopulation, a higher general (microsatellite) heterozygosity (P = 2x10^-4^) was found in this group compared to its original Mt. Kulal subpopulation. Microsatellite loci themselves are not likely to underlie the heterozygosity advantage. Due to high numbers of polymorphic variants/haplotypes combined with low numbers of dogs available, it was impossible to make such an estimate for the MHC region, the prime candidate for testing the heterozygosity advantage. However, such a comparison could be made for the non-MHC IR gene markers studied, and no specific differences were observed in the parameters of overall genetic diversity. Nevertheless, comparisons of allelic frequencies in specific loci associated with infections in this population [47] showed significant differences. For the gene *LY96*, differences were observed not only between surviving and original Mt. Kulal dogs (Table 6), but also between the Kenyan and European groups. Significantly (P = 1,81x10^-6^) lower frequencies of a resistance-associated *Ly96* allele were observed in European dogs, while significantly higher frequencies of this allele were found in the “survivor” group. Similarly, differences between Kenyan and Czech dogs (P = 1x10^-4^) as well between the survivor group and the Kenyan group as a whole (P = 0.023) were found for *MYD88*. These data support the assumption that at least some markers associated with infection in this population might have an adaptive value.

The increased heterozygosity in microsatellite markers observed in survivors difference suggests a higher overall genomic diversity associated with higher age under these particular conditions. Similar or even identical panels of microsatellite markers were used for assessing the extent of neutral and/or non-neutral genetic diversity in dogs and wolves [20, 76]. Therefore, the interpretation that in this group, the survival of its members could be influenced by the overall genomic heterozygosity rather than by particular genotypes/haplotypes in specific loci seems to be biologically plausible. On the other hand, effects of a progressive increase of homozygosity, where older generations are more heterozygous, could also be an alternative explanation. However, it seems that more generations would be necessary for a significant change.

## Conclusions

Taken together, it seems that the genetic structure of this particular population of Kenyan village dogs is mostly determined by geographical and anthropogenic factors influencing the gene flow between various subpopulations rather than by biological factors, such as genetic contribution of original migrating populations and/or the pathogen-mediated selection. On the other hand, the study of oldest surviving dogs suggested a biological mechanism, i.e. a possible advantage of the overal heterozygosity marked by the the microsatellite loci analyzed. Adaptation to a putative pathogen pressure in this pathogen-rich environment seems to be more difficult to detect. To our knowledge, this is the first report on the genetic diversity in non-MHC IR candidate gene markers in domestic dogs. Our data show that polymorphic candidate non-MHC IR gene markers are a rather heterogeneous group in terms of their value for estimating non-neutral genetic diversity. Differences between populations in disease-associated markers suggest that a panel of selected polymorphisms associated with diseases in the particular population could be a way to detect subtle effects of pathogen-driven selection, but that a higher number of markers and their careful selection for a particular purpose would be necessary.

## Supporting information

S1 Fig Graphical representation of the estimated membership of the Kenyan and European dogs in each of the inferred clusters (K = 3) based on microsatellite markers. (TIF)

S2 Fig Graphical representation of the estimated membership of the Kenyan and European dogs in each of the inferred clusters (K = 3) based on SNP markers. (TIF)

S3 Fig Estimation of the population substructure across MHC loci in the Kenyan village dogs (K = 3) using Structure software. (TIF)

S4 Fig Principal coordinates analysis (PCoA) (first and second coordinate) of survivor (n = 21) and Mt. Kulal (n = 39) dogs based on microsatellite markers. (TIF)

S5 Fig Principal coordinates analysis (PCoA) (first and second coordinate) of survivor (n = 21) and Mt. Kulal dogs (n = 39) based on SNP markers. (TIF)

S1 Table The allelic frequencies of 27 microsatellite loci for Kenyan (n = 150) and European dogs (n = 68). (DOCX)

S2 Table Observed and expected heterozygosities and observed number of alleles for 27 loci of microsatellite for Kenyan dogs, European dogs and Survivor dogs. SEM standard error of the mean.
(DOCX)

S3 Table Number of alleles per locus. number of effective alleles per locus and fixation index for microsatellite markers in Kenyan village dog subpopulations and European dogs. MK–Mt. Kulal. MN–Mt. Ngyiro. LK–Lake Turkana. E–European dogs. SEM standard error of the mean.
(DOCX)

S4 Table The allelic frequencies of 16SNP markers for Kenyan dogs subpopulations, entire Kenyan dogs population and European dogs. (DOCX)

S5 Table Observed, expected heterozygosities and number of genotypes for 16 SNP loci for Kenyan dogs. European dogs and Survivor dogs. * Not the same alleles. ** only AA and Aa genotypes.
(DOCX)

S6 Table The allelic frequencies of 27 microsatellite loci for Kenyan dogs subpopulations (n = 50 Mt. Kulal dogs, 50 Mt. Ngyiro dogs, 50 Lake Turkana dogs). (DOCX)

S7 Table Observed and expected heterozygosities for 27 microsatellie loci for Kenyan dogs subpopulations. MK- Mt. Kulal. MN- Mt. Ngyiro. LK- Lake Turkana.
(DOCX)

S8 Table Fixation indices over all Kenyan village dogs (n = 150) for microsatellite markers. (DOCX)

S9 Table The allelic frequencies of MHC loci for Kenyan dogs subpopulations (n = 41 Mt. Kulal dogs, 47 Mt. Ngyiro dogs, 47 Lake Turkana dogs). (DOCX)

S10 Table Observed. expected heterozygosities and number of alleles for MHC loci for Kenyan village dogs subpopulations. (DOCX)

S11 Table Observed. expected heterozygosities. number of genotypes and MAF for 16 SNP loci for Kenyan dogs subpopulations. MK- Mt. Kulal. MN- Mt. Ngyiro. LK- Lake Turkana* Not the same alleles. ** only AA and Aa genotypes.
(DOCX)

S12 Table The correlation of Reynolds genetic distances for three types of markers. ^NS^ Non-significant, **P<0.01.
(DOCX)

S13 Table Molecular diversity of microsatellites: Number of alleles. observed (Ho) and expected (He) heterozygosities and loci out of HWE.
(DOCX)

S14 Table Observed and expected heterozygosities, observed number of alleles and number of loci out of HWE for 10 microsatellite loci (FHC2010, FHC2054, FHC2079, PEZ1, PEZ3, PEZ5, PEZ6, PEZ8, PEZ12, PEZ20) for Kenyan dogs, European dogs (group of 24 pure breeds and 6 mongrels) and four groups of pure breeds- Basset, Bernese Mountain Dog, European Terrier and Caucasian shepherd dog. 1* monomorphic locus.
(DOCX)

S15 Table Tajima´s test of selective neutrality. (DOCX)
