# Supplementary figures and images for: Genetic diversity and population structure of African village dogs based on microsatellite and immunity-related molecular markers
Source: PLoS One. 2018 Jun 25;13(6):e0199506. doi: 10.1371/journal.pone.0199506 (PMC6016929; doi:10.1371/journal.pone.0199506)

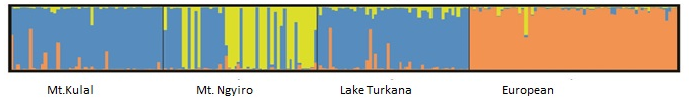

Supplement: S1 Fig — (TIF) [file pone.0199506.s001.tif]

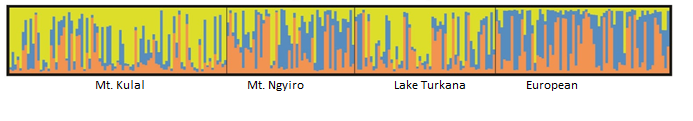

Supplement: S2 Fig — (TIF) [file pone.0199506.s002.tif]

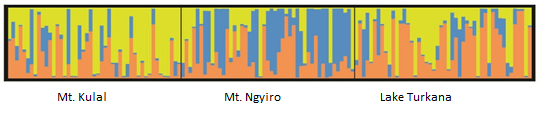

Supplement: S3 Fig — (TIF) [file pone.0199506.s003.tif]

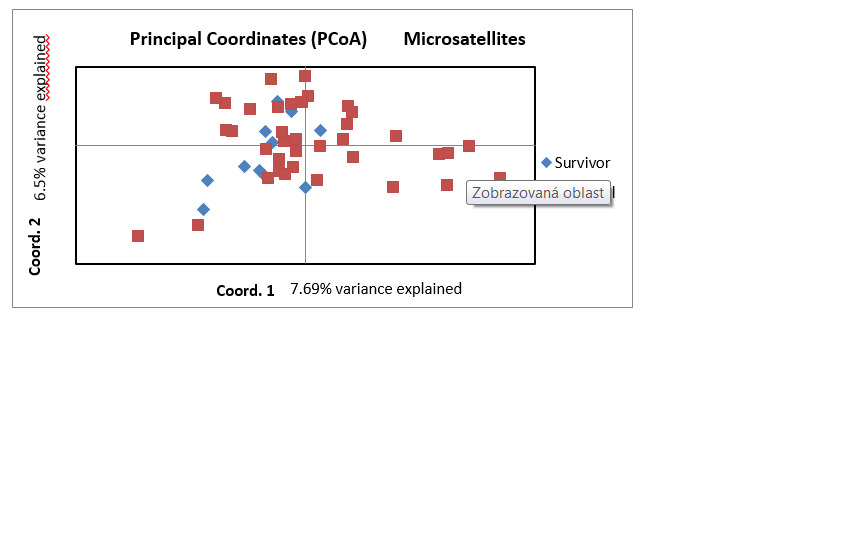

Supplement: S4 Fig — (TIF) [file pone.0199506.s004.tif]

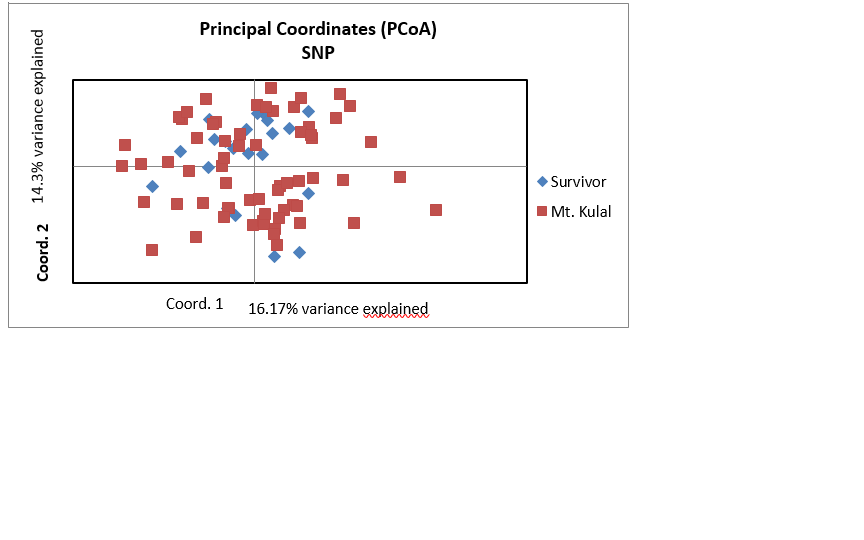

Supplement: S5 Fig — (TIF) [file pone.0199506.s005.tif]
