# Supplementary material for: Genetic diversity and population structure of African village dogs based on microsatellite and immunity-related molecular markers
Source: PLoS One. 2018 Jun 25;13(6):e0199506. doi: 10.1371/journal.pone.0199506 (PMC6016929; doi:10.1371/journal.pone.0199506)
Supplement: S1 Table — (DOCX) [file pone.0199506.s006.docx]

| **FHC2010**  **CFA24** | **FHC2054**  **CFA12** | **FHC2079**  **CFA24** | **PEZ1**  **CFA7** | **PEZ12**  **CFA3** |
| --- | --- | --- | --- | --- |
| Allele Kenyan European  219 0.01779 0.01471  223 0.08897 0.07353  227 0.24199 0.43382  231 0.27046 0.16176  235 0.36299 0.27206  239 0.00712 0.04412  243 0.01068 0.00000 | Allele Kenyan European  143 0.00000 0.01471  147 0.00000 0.06618  148 0.00352 0.00000  151 0.07746 0.22794  152 0.03169 0.00000  155 0.12676 0.21324  156 0.15845 0.00000  159 0.05634 0.06618  160 0.02817 0.00000  163 0.08803 0.11029  164 0.06338 0.00000  167 0.08451 0.14706  168 0.05986 0.00000  171 0.05282 0.10294  172 0.04577 0.00000  175 0.06338 0.05147  176 0.05986 0.00000 | Allele Kenyan European  261 0.00000 0.00735  269 0.26736 0.33824  273 0.23264 0.33824  277 0.13889 0.19118  281 0.10764 0.00000  283 0.00347 0.00000  285 0.03819 0.03676  289 0.03819 0.05882  293 0.16667 0.02941  297 0.00694 0.00000 | Allele Kenyan European  106 0.26148 0.05147  110 0.11307 0.12500  114 0.27562 0.27941  118 0.22615 0.30147  122 0.12367 0.16912  126 0.00000 0.04412  130 0.00000 0.02941 | Allele Kenyan European  256 0.00000 0.02941  260 0.03887 0.10294  264 0.20495 0.08088  268 0.21908 0.23529  272 0.18021 0.23529  276 0.08481 0.15441  280 0.12367 0.07353  284 0.05300 0.03676  288 0.02827 0.01471  292 0.02473 0.00000  296 0.01767 0.01471  300 0.01060 0.00735  304 0.01413 0.00735  308 0.00000 0.00735 |
| **PEZ20**  **unmapped** | **PEZ3**  **CFA19** | **PEZ5**  **CFA12** | **PEZ6**  **CFA27** | **PEZ8**  **CFA17** |
| Allele Kenyan European  167 0.01859 0.00000  171 0.20074 0.08824  175 0.31599 0.52206  179 0.20446 0.25735  183 0.14498 0.11029  187 0.01115 0.01471  191 0.02230 0.00735  194 0.01487 0.00000  195 0.06691 0.00000 | Allele Kenyan European  108 0.00000 0.02941  111 0.00000 0.00735  114 0.00360 0.01471  117 0.07914 0.10294  120 0.06115 0.12500  123 0.15108 0.21324  126 0.20504 0.15441  129 0.05755 0.13235  132 0.04317 0.04412  135 0.11871 0.08088  138 0.06115 0.06618  141 0.12950 0.01471  150 0.00360 0.00000  93 0.08633 0.01471 | Allele Kenyan European  102 0.40989 0.59559  106 0.22615 0.11765  110 0.27915 0.23529  114 0.02120 0.02206  94 0.02827 0.02941  98 0.03534 0.00000 | Allele Kenyan European  168 0.02787 0.00000  170 0.00348 0.00000  172 0.07317 0.11765  174-1 0.00000 0.00735  176 0.14634 0.12500  180 0.13937 0.26471  182 0.00348 0.00000  184 0.25436 0.24265  186 0.04530 0.00000  188 0.23345 0.13235  192 0.03484 0.08088  196 0.03136 0.02206  200 0.00697 0.00735 | Allele Kenyan European  211 0.00000 0.00735  215 0.00000 0.02206  219 0.02837 0.02941  223 0.21631 0.16176  227 0.18440 0.17647  231 0.21986 0.27941  235 0.23404 0.16912  239 0.10284 0.12500  243 0.01418 0.02206  247 0.00000 0.00735 |
| **AHTk211**  **CFA26** | **CXX279**  **CFA22** | **INU055**  **CFA10** | **REN169O18**  **CFA29** | **REN54P11**  **CFA18** |
| Allele Kenyan European  85 0.00333 0.00000  87 0.41333 0.27941  89 0.08333 0.13235  91 0.40000 0.38971  93 0.01000 0.03676  95 0.09000 0.13971  97 0.00000 0.02206 | Allele Kenyan European  114 0.01010 0.00735 116 0.07744 0.11029  118 0.37037 0.30147  120 0.01010 0.12500  122 0.03367 0.01471  124 0.04377 0.20588  126 0.31650 0.11765  128 0.13131 0.04412  130 0.00000 0.05147  132 0.00673 0.02206 | Allele Kenyan European  200 0.08621 0.00000  208 0.01724 0.02206  210 0.49310 0.38235  212 0.02069 0.13235  214 0.07931 0.16912  216 0.00345 0.04412  218 0.02759 0.14706  220 0.24483 0.06618  222 0.01379 0.02941  224 0.00690 0.00735  226 0.00690 0.00000 | Allele Kenyan European  156 0.00000 0.01471  158 0.00000 0.02941  160 0.00000 0.05147  162 0.16054 0.37500  164 0.19732 0.13235  166 0.29097 0.10294  168 0.22408 0.12500  170 0.07692 0.16912  172 0.05017 0.00000 | Allele Kenyan European  222 0.08081 0.05147  226 0.10774 0.25735  228 0.03030 0.02941  230 0.00337 0.00735  232 0.01684 0.27941  234 0.47811 0.16912  236 0.08754 0.11765  238 0.10101 0.07353  240 0.04714 0.00735  242 0.02694 0.00735  244 0.01684 0.00000  246 0.00337 0.00000 |
| **AHT137**  **CFA1116** | **AHTh260**  **CFA** | **AHTk253**  **CFA23** | **INRA21**  **CFA21** | **REN169D01**  **CFA14** |
| Allele Kenyan European  131 0.09091 0.20741  133 0.04714 0.04444  135 0.16498 0.05185  137 0.06061 0.13333  139 0.01684 0.01481  141 0.00000 0.03704  143 0.02020 0.04444  145 0.03367 0.05926  147 0.24579 0.24444  149 0.19192 0.03704  151 0.07744 0.11111  153 0.05051 0.01481 | Allele Kenyan European  234 0.00000 0.00735  235 0.00000 0.01471  236 0.00667 0.00000  238 0.04000 0.19118  240 0.04000 0.06618  242 0.23667 0.04412  244 0.10333 0.11029  246 0.36667 0.25000  248 0.15000 0.05147  250 0.02333 0.07353  252 0.03333 0.09559  254 0.00000 0.08088  256 0.00000 0.01471 | Allele Kenyan European  280 0.00673 0.01471  282 0.04714 0.00735  284 0.06734 0.05882  286 0.21212 0.14706  288 0.38384 0.30882  290 0.11111 0.25735  292 0.13468 0.16176  294 0.00337 0.03676  296 0.01347 0.00735  298 0.02020 0.00000 | Allele Kenyan European  101 0.11000 0.31852  103 0.01000 0.03704  105 0.11000 0.03704  107 0.00333 0.00000  109 0.00667 0.00000  115 0.00333 0.00000  91 0.00667 0.02963  95 0.41667 0.31852  97 0.17333 0.08148  99 0.16000 0.17778 | Allele Kenyan European  202 0.08667 0.09559  208 0.01667 0.00000  210 0.05333 0.08824  212 0.05000 0.22794  214 0.01000 0.03676  216 0.61333 0.27206  218 0.02667 0.08088  220 0.13667 0.14706  222 0.00667 0.05147 |
| **AHT121**  **CFA13** | **AHTh171**  **CFA06** | **REN162C04**  **CFA07** | **REN247M23**  **CFA15** | **FHC2848**  **CFA02** |
| Allele Kenyan European  100 0.13131 0.16541  102 0.10774 0.15789  104 0.10438 0.09023  106 0.06061 0.12030  108 0.01010 0.09774  110 0.01010 0.00752  112 0.02357 0.01504  114 0.00673 0.01504  72 0.00000 0.00752  86 0.00673 0.00000  88 0.00000 0.00752  90 0.09428 0.00000  92 0.04714 0.05263  94 0.05387 0.06015  96 0.14815 0.10526  98 0.19529 0.09774 | Allele Kenyan European  214 0.00685 0.00000  217 0.15753 0.01471  219 0.07192 0.23529  221 0.28425 0.05147  223 0.15411 0.01471  225 0.12329 0.21324  227 0.01370 0.05147  229 0.04795 0.11029  231 0.02740 0.03676  233 0.08219 0.14706  235 0.00000 0.09559  237 0.03082 0.02941 | Allele Kenyan European  192 0.01027 0.00735  194 0.02055 0.00735  196 0.00685 0.01471  198 0.03425 0.00000  200 0.03425 0.04412  202 0.05137 0.22794  204 0.40753 0.20588  206 0.36986 0.29412  208 0.04795 0.10294  210 0.01712 0.05147  212 0.00000 0.04412 | Allele Kenyan European  266 0.00685 0.00735  268 0.58904 0.46324  270 0.13356 0.17647  272 0.14726 0.24265  274 0.00000 0.07353  276 0.02397 0.00000  278 0.06507 0.03676  280 0.03082 0.00000  282 0.00342 0.00000 | Allele Kenyan European  228 0.01375 0.00000  230 0.07904 0.05303  232 0.00344 0.03788  234 0.00344 0.07576  236 0.31271 0.09091  238 0.16838 0.28030  240 0.29210 0.28788  242 0.04124 0.06061  244 0.02749 0.05303  246 0.04811 0.04545  248 0.01031 0.00758  252 0.00000 0.00758 |
| **INU005**  **CFA33** | **INU030**  **CFA12** |  |  |  |
| Allele Kenyan European  106 0.00000 0.00735  110 0.01010 0.00735  112 0.05724 0.12500  114 0.00337 0.00000  122 0.00337 0.08824  124 0.10438 0.33088  126 0.40741 0.24265  128 0.09764 0.10294  130 0.18182 0.03676  132 0.13468 0.05882 | Allele Kenyan European  144 0.06122 0.26471  146 0.34354 0.02941  148 0.07483 0.04412  150 0.37075 0.44853  152 0.02041 0.14706  154 0.12585 0.05147  156 0.00340 0.01471 |  |  |  |
