# Supplementary material for: Genetic diversity and population structure of African village dogs based on microsatellite and immunity-related molecular markers
Source: PLoS One. 2018 Jun 25;13(6):e0199506. doi: 10.1371/journal.pone.0199506 (PMC6016929; doi:10.1371/journal.pone.0199506)
Supplement: S2 Table — SEM standard error of the mean. (DOCX) [file pone.0199506.s007.docx]

|  | Chrom. | Observed heterozygosity | | | Expected heterozygosity | | | Number of observed alleles | | | P- value | | |
| --- | --- | --- | --- | --- | --- | --- | --- | --- | --- | --- | --- | --- | --- |
|  |  |  |  |  |  |  |  |  |  |  |  |  |  |
|  |  | Kenyan dogs  (n=150) | European dogs  (n=68) | Survivor dogs  (n=21) | Kenyan dogs  (n=150) | European dogs  (n=68) | Survivor dogs  (n=21) | Kenyan dogs  (n=150) | European dogs  (n=68) | Survivor dogs  (n=21) | Kenyan dogs | European dogs | Survivor dogs |
| FH2010 | CFA24 | 0.619 | 0.500 | 0.810 | 0.731 | 0.709 | 0.664 | 7 | 6 | 5 | 0.0469 | 0.0001 | 0.0415 |
| FH2054 | CFA12 | 0.773 | 0.676 | 0.900 | 0.916 | 0.853 | 0.856 | 15 | 9 | 13 | 0.0000 | 0.0146 | 0.6562 |
| FH2079 | CFA24 | 0.762 | 0.588 | 0.667 | 0.816 | 0.734 | 0.775 | 9 | 7 | 6 | 0.0886 | 0.0031 | 0.5070 |
| PEZ1 | CFA7 | 0.707 | 0.721 | 0.810 | 0.780 | 0.787 | 0.750 | 5 | 7 | 5 | 0.0235 | 0.0155 | 0.6772 |
| PEZ12 | CFA3 | 0.773 | 0.691 | 0.952 | 0.852 | 0.846 | 0.871 | 12 | 13 | 10 | 0.0127 | 0.0163 | 0.5302 |
| PEZ20 | unmapped | 0.707 | 0.559 | 0.619 | 0.794 | 0.646 | 0.702 | 9 | 6 | 6 | 0.0116 | 0.0845 | 0.4793 |
| PEZ3 | CFA19 | 0.803 | 0.809 | 0.952 | 0.881 | 0.879 | 0.883 | 12 | 13 | 10 | 0.0587 | 0.3692 | 0.6814 |
| PEZ5 | CFA12 | 0.629 | 0.456 | 0.810 | 0.703 | 0.579 | 0.662 | 6 | 5 | 5 | 0.0173 | 0.1794 | 0.6570 |
| PEZ6 | CFA27 | 0.704 | 0.647 | 0.762 | 0.832 | 0.823 | 0.800 | 12 | 9 | 9 | 0.0016 | 0.0000 | 0.4467 |
| PEZ8 | CFA17 | 0.734 | 0.647 | 0.900 | 0.807 | 0.825 | 0.797 | 7 | 10 | 6 | 0.6306 | 0.0031 | 0.0528 |
| AHTk211 | CFA26 | 0.560 | 0.544 | 0.524 | 0.656 | 0.737 | 0.684 | 6 | 6 | 6 | 0.0075 | 0.0034 | 0.0297 |
| CXX279 | CFA22 | 0.716 | 0.662 | 0.810 | 0.739 | 0.826 | 0.712 | 9 | 10 | 6 | 0.5584 | 0.0111 | 0.5470 |
| INU055 | CFA10 | 0.507 | 0.559 | 0.762 | 0.684 | 0.784 | 0.775 | 10 | 9 | 8 | 0.0000 | 0.0000 | 0.4539 |
| REN169O18 | CFA29 | 0.725 | 0.676 | 0.810 | 0.795 | 0.789 | 0.799 | 6 | 8 | 6 | 0.1529 | 0.2287 | 0.9452 |
| REN54P11 | CFA18 | 0.701 | 0.603 | 0.762 | 0.733 | 0.810 | 0.758 | 12 | 10 | 8 | 0.8321 | 0.0053 | 0.9427 |
| AHT137 | CFA11 | 0.845 | 0.672 | 0.850 | 0.854 | 0.860 | 0.855 | 11 | 12 | 8 | 0.0268 | 0.0005 | 0.4589 |
| AHTh260 | CFA16 | 0.753 | 0.647 | 0.762 | 0.774 | 0.865 | 0.728 | 9 | 12 | 6 | 0.4808 | 0.0192 | 0.8534 |
| AHTk253 | CFA23 | 0.735 | 0.706 | 0.905 | 0.772 | 0.791 | 0.858 | 10 | 9 | 9 | 0.0272 | 0.2182 | 0.4045 |
| INRA21 | CFA21 | 0.673 | 0.687 | 0.762 | 0.749 | 0.761 | 0.827 | 10 | 7 | 7 | 0.2975 | 0.1251 | 0.3969 |
| REN169DO1A | CFA14 | 0.500 | 0.574 | 0.619 | 0.593 | 0.831 | 0.685 | 9 | 8 | 7 | 0.0009 | 0.0000 | 0.3569 |
| AHT121 | CFA13 | 0.789 | 0.769 | 0.857 | 0.885 | 0.895 | 0.873 | 14 | 14 | 8 | 0.0531 | 0.0295 | 0.3093 |
| AHTh171 | CFA06 | 0.634 | 0.765 | 0.619 | 0.842 | 0.855 | 0.760 | 11 | 11 | 7 | 0.0000 | 0.0518 | 0.1969 |
| REN162CO4 | CFA07 | 0.637 | 0.677 | 0.700 | 0.691 | 0.808 | 0.645 | 10 | 10 | 5 | 0.5151 | 0.0438 | 0.1585 |
| REN247M23 | CFA15 | 0.531 | 0.459 | 0.476 | 0.610 | 0.694 | 0.561 | 8 | 6 | 5 | 0.0994 | 0.0003 | 0.0659 |
| FHC2848 | CFA02 | 0.690 | 0.621 | 0.857 | 0.780 | 0.818 | 0.810 | 11 | 11 | 7 | 0.0076 | 0.0000 | 0.0456 |
| INU005 | CFA33 | 0.669 | 0.632 | 0.850 | 0.762 | 0.799 | 0.806 | 9 | 9 | 6 | 0.0073 | 0.0438 | 0.2122 |
| INU030 | CFA12 | 0.644 | 0.632 | 0.714 | 0.721 | 0.707 | 0.750 | 7 | 7 | 6 | 0.1969 | 0.6929 | 0.5091 |
| Mean |  | | | | | | | 9.481 | 9.037 | 7.037 |  | | |
| SEM |  |  |  |  |  |  |  | 0.480 | 0.470 | 0.341 |  |  |  |

SEM standard error of the mean
