# Supplementary material for: Genetic diversity and population structure of African village dogs based on microsatellite and immunity-related molecular markers
Source: PLoS One. 2018 Jun 25;13(6):e0199506. doi: 10.1371/journal.pone.0199506 (PMC6016929; doi:10.1371/journal.pone.0199506)
Supplement: S3 Table — MK–Mt. Kulal. MN–Mt. Ngyiro. LK–Lake Turkana. E–European dogs. SEM standard error of the mean. (DOCX) [file pone.0199506.s008.docx]

| Locus | Number of alleles per locus | | | | | | | | Fixation index | | | |
| --- | --- | --- | --- | --- | --- | --- | --- | --- | --- | --- | --- | --- |
|  | Total | | | | Effective | | | |  |  |  |  |
|  | (n= 50) | (n= 50) | (n= 50) | (n = 68) | (n= 50) | (n= 50) | (n= 50) | (n = 68) | (n= 50) | (n= 50) | (n= 50) | (n = 68) |
|  | MK | MN | LT | E | MK | MN | LT | E | MK | MN | LT | E |
| FHC2010 | 5 | 4 | 5 | 6 | 3.14 | 3.51 | 3.67 | 3.38 | 0.12 | 0.21 | 0.04 | 0.29 |
| FHC2054 | 14 | 14 | 14 | 9 | 9.80 | 10.77 | 7.68 | 6.52 | 0.05 | 0.21 | 0.14 | 0.20 |
| FHC2079 | 8 | 7 | 8 | 7 | 5.27 | 4.66 | 4.64 | 3.69 | 0.01 | 0.07 | 0.04 | 0.19 |
| PEZ1 | 5 | 5 | 5 | 7 | 3.47 | 3.95 | 4.64 | 4.57 | 0.10 | 0.06 | -0.01 | 0.08 |
| PEZ12 | 11 | 10 | 11 | 13 | 7.07 | 4.14 | 6.55 | 6.25 | 0.12 | 0.01 | 0.04 | 0.18 |
| PEZ20 | 8 | 7 | 8 | 6 | 3.68 | 4.96 | 4.57 | 2.79 | 0.13 | 0.05 | 0.06 | 0.13 |
| PEZ3 | 11 | 9 | 10 | 13 | 8.27 | 6.19 | 5.72 | 7.84 | 0.01 | 0.10 | 0.07 | 0.07 |
| PEZ5 | 6 | 5 | 5 | 5 | 3.55 | 2.65 | 3.33 | 2.35 | 0.07 | 0.13 | 0.03 | 0.21 |
| PEZ6 | 9 | 10 | 10 | 9 | 5.09 | 5.32 | 5.21 | 5.46 | 0.25 | 0.12 | 0.01 | 0.21 |
| PEZ8 | 7 | 7 | 6 | 10 | 5.23 | 4.32 | 4.52 | 5.51 | 0.02 | 0.12 | 0.04 | 0.21 |
| AHTk211 | 6 | 5 | 4 | 6 | 3.14 | 2.33 | 2.88 | 3.72 | 0.30 | 0.05 | -0.01 | 0.26 |
| CXX279 | 8 | 7 | 7 | 10 | 3.83 | 3.76 | 3.50 | 5.55 | 0.12 | -0.03 | -0.04 | 0.19 |
| INU055 | 9 | 5 | 7 | 9 | 3.24 | 1.86 | 4.20 | 4.51 | 0.29 | 0.29 | 0.08 | 0.28 |
| REN169O18 | 6 | 6 | 6 | 8 | 4.46 | 4.34 | 4.88 | 4.62 | 0.07 | 0.18 | -0.03 | 0.14 |
| REN54P11 | 12 | 9 | 9 | 10 | 3.54 | 3.19 | 3.96 | 5.12 | -0.03 | 0.07 | 0.04 | 0.25 |
| AHT137 | 9 | 11 | 11 | 12 | 7.062 | 6.57 | 4.24 | 6.75 | -0.05 | 0.01 | -0.05 | 0.21 |
| AHTh260 | 7 | 7 | 9 | 12 | 3.31 | 4.91 | 4.44 | 7.05 | 0.03 | 0.07 | -0.08 | 0.25 |
| AHTk253 | 9 | 9 | 9 | 9 | 4.74 | 4.24 | 3.44 | 4.66 | -0.09 | 0.14 | 0.04 | 0.10 |
| INRA21 | 8 | 6 | 8 | 7 | 4.48 | 2.59 | 4.44 | 4.04 | 0.15 | 0.17 | -0.11 | 0.09 |
| REN169D01 | 8 | 7 | 8 | 8 | 2.93 | 2.07 | 2.24 | 5.71 | 0.18 | 0.07 | 0.13 | 0.31 |
| AHT121 | 11 | 10 | 13 | 14 | 6.93 | 7.08 | 8.56 | 8.78 | 0.03 | 0.24 | 0.01 | 0.13 |
| AHTh171 | 9 | 7 | 10 | 11 | 5.41 | 5.37 | 6.16 | 6.59 | 0.24 | 0.41 | 0.05 | 0.10 |
| REN162C04 | 10 | 7 | 10 | 10 | 3.16 | 2.87 | 3.43 | 5.04 | 0.10 | 0.12 | -0.02 | 0.16 |
| REN247M23 | 5 | 7 | 7 | 6 | 2.70 | 2.19 | 2.67 | 3.21 | 0.22 | 0.12 | 0.01 | 0.34 |
| FH2848 | 10 | 7 | 8 | 11 | 3.89 | 3.72 | 4.43 | 5.31 | 0.01 | 0.16 | 0.09 | 0.24 |
| INU005 | 6 | 8 | 7 | 9 | 4.10 | 3.78 | 4.22 | 4.82 | 0.09 | 0.29 | -0.05 | 0.20 |
| INU030 | 6 | 6 | 6 | 7 | 3.21 | 3.18 | 3.23 | 3.35 | 0.17 | 0.04 | -0.01 | 0.10 |
| Mean | 8.26 | 7.37 | 8.19 | 9.04 | 4.62 | 4.24 | 4.50 | 5.08 | 0.10 | 0.13 | 0.02 | 0.19 |
| SEM | 0.44 | 0.43 | 0.47 | 0.47 | 0.35 | 0.36 | 0.28 | 0.30 | 0.02 | 0.02 | 0.01 | 0.01 |

MK – Mt. Kulal. MN – Mt. Ngyiro. LK – Lake Turkana. E – European dogs. SEM standard error of the mean
