# Supplementary material for: Genetic diversity and population structure of African village dogs based on microsatellite and immunity-related molecular markers
Source: PLoS One. 2018 Jun 25;13(6):e0199506. doi: 10.1371/journal.pone.0199506 (PMC6016929; doi:10.1371/journal.pone.0199506)
Supplement: S4 Table — (DOCX) [file pone.0199506.s009.docx]

| SNP marker | Restriction enzyme | allele | Mt. Kulal  (n=50) | Mt. Ngyiro  (n=50) | Lake Turkana  (n=50) | Kenyan  (n= 150) | European  (n=68) |
| --- | --- | --- | --- | --- | --- | --- | --- |
| *NOS3/*a | HpyCH4V | A | 0.97 | 0.87 | 0.98 | 0.94 | 0.76 |
|  |  | T | 0.03 | 0.13 | 0.02 | 0.06 | 0.24 |
| *NOS3*/b | BsaJI | A | 0.78 | 0.65 | 0.76 | 0.73 | 0.73 |
|  |  | C | 0.22 | 0.35 | 0.24 | 0.27 | 0.27 |
| *IL6*/a | BseYI | A | 0.97 | 0.92 | 0.99 | 0.96 | 0.95 |
|  |  | G | 0.03 | 0.08 | 0.01 | 0.04 | 0.05 |
| *IL6*/b | HpaI | C | 0.17 | 0.14 | 0.03 | 0.11 | 0.23 |
|  |  | T | 0.83 | 0.86 | 0.97 | 0.89 | 0.77 |
| *TLR1* | Sau96I | C | 0.89 | 0.61 | 0.70 | 0.74 | 0.65 |
|  |  | T | 0.11 | 0.39 | 0.30 | 0.26 | 0.35 |
| *TLR2* | NspI | A | 0.03 | 0.07 | 0.02 | 0.04 | 0.13 |
|  |  | C | 0.97 | 0.93 | 0.98 | 0.96 | 0.87 |
| *TLR4*/a | XcmI | A | 0.33 | 0.51 | 0.46 | 0.43 | 0.48 |
|  |  | G | 0.67 | 0.49 | 0.54 | 0.57 | 0.52 |
| *TLR4*/b | HinPI | C | 0.57 | 0.28 | 0.46 | 0.44 | 0.24 |
|  |  | G | 0.43 | 0.72 | 0.54 | 0.56 | 0.76 |
| *TLR7* | BtsCI | C | 0.43 | 0.56 | 0.38 | 0.46 | 0.78 |
|  |  | A | 0.57 | 0.44 | 0.62 | 0.54 | 0.22 |
| *TLR9*/a | NgoMIV | A | 0.87 | 0.89 | 0.71 | 0.82 | 0.61 |
|  |  | G | 0.13 | 0.11 | 0.29 | 0.18 | 0.39 |
| *TLR9*/b | BseYI | A | 0.68 | 0.66 | 0.75 | 0.70 | 0.45 |
|  |  | G | 0.32 | 0.34 | 0.25 | 0.30 | 0.55 |
| *LY96*/a | BtsCI | C | 0.85 | 0.64 | 0.73 | 0.74 | 0.50 |
|  |  | T | 0.15 | 0.36 | 0.27 | 0.26 | 0.50 |
| *LY96*/b | Hpy166II | A | 0.71 | 0.43 | 0.57 | 0.57 | 0.32 |
|  |  | G | 0.29 | 0.57 | 0.43 | 0.43 | 0.68 |
| *MYD88*/a | BglI | C | 0.50 | 0.47 | 0.53 | 0.50 | 0.28 |
|  |  | T | 0.50 | 0.53 | 0.47 | 0.50 | 0.72 |
| *MYD88/*b | BamHI | C | 0.48 | 0.50 | 0.51 | 0.50 | 0.50 |
|  |  | G | 0.52 | 0.50 | 0.49 | 0.50 | 0.50 |
| *MYD88*/c | ApaLI | C | 0.78 | 0.80 | 0.74 | 0.77 | 0.78 |
|  |  | T | 0.22 | 0.20 | 0.26 | 0.23 | 0.24 |
