# Supplementary material for: Genetic diversity and population structure of African village dogs based on microsatellite and immunity-related molecular markers
Source: PLoS One. 2018 Jun 25;13(6):e0199506. doi: 10.1371/journal.pone.0199506 (PMC6016929; doi:10.1371/journal.pone.0199506)
Supplement: S5 Table — * Not the same alleles. ** only AA and Aa genotypes. (DOCX) [file pone.0199506.s010.docx]

|  |  | Observed heterozygosity | | | Expected heterozygosity | | | P- value | | | Number of genotypes | | |
| --- | --- | --- | --- | --- | --- | --- | --- | --- | --- | --- | --- | --- | --- |
|  |  |  |  |  |  |  |  |  |  |  |  |  |  |
|  |  | Kenyan dogs  (n=150) | European dogs  (n=68) | Survivor dogs  (n=21) | Kenyan dogs  (n=150) | European dogs  (n=68) | Survivor dogs  (n=21) | Kenyan dogs | European dogs | Survivor dogs | Kenyan dogs  (n=150) | European dogs  (n=68) | Survivor dogs  (n=21) |
|  |  |  |  |  |  |  |  |  |  |  |  |  |  |
| *NOS3* | a | 0.107 | 0.309 | 0.048 | 0.113 | 0.370 | 0.048 | 0.4148 | 0.1915 | 1.0000 | 3 | 3 | 2** |
|  | b | 0.367 | 0.309 | 0.381 | 0.396 | 0.399 | 0.372 | 0.4120 | 0.0739 | 1.0000 | 3 | 3 | 3 |
| *IL6* | a | 0.081 | 0.074 | 0.050 | 0.078 | 0.098 | 0.050 | 1.0000 | 0.1514 | 1.0000 | 2** | 3 | 2** |
|  | b | 0.188 | 0.309 | 0.429 | 0.203 | 0.355 | 0.396 | 0.4063 | 0.3115 | 1.0000 | 3 | 3 | 3 |
| *TLR1* |  | 0.338 | 0.191 | 0.190 | 0.389 | 0.456 | 0.251 | 0.1333 | 0.0000 | 0.3366 | 3 | 3 | 3 |
| *TLR2* |  | 0.080 | 0.147 | mono | 0.077 | 0.231 | mono | 1.0000 | 0.0111 | mono | 2** | 3 | 1 |
| *TLR4* | a | 0.467 | 0.397 | 0.619 | 0.493 | 0.503 | 0.508 | 0.6088 | 0.0926 | 0.3927 | 3 | 3 | 3 |
|  | b | 0.433 | 0.338 | 0.650 | 0.494 | 0.370 | 0.512 | 0.1419 | 0.5102 | 0.3681 | 3 | 3 | 3 |
| *TLR7* |  | 0.134 | 0.147 | 0.190 | 0.498 | 0.346 | 0.372 | 0.0000 | 0.0000 | 0.0461 | 3 | 3 | 3 |
| *TLR9* | a | 0.273 | 0.338 | 0.333 | 0.292 | 0.479 | 0.285 | 0.4056 | 0.0206 | 1.0000 | 3 | 3 | 3 |
|  | b | 0.393 | 0.299 | 0.524 | 0.424 | 0.498 | 0.494 | 0.4305 | 0.0015 | 1.0000 | 3 | 3 | 3 |
| *LY96* | a | 0.366 | 0.353 | 0.200 | 0.385 | 0.504 | 0.185 | 0.6636 | 0.0182 | 1.0000 | 3 | 3 | 2** |
|  | b | 0.497 | 0.294 | 0.381 | 0.492 | 0.441 | 0.316 | 1.0000 | 0.0117 | 1.0000 | 3 | 3 | 2** |
| *MYD88* | a | 0.493 | 0.382 | 0.571 | 0.502 | 0.406 | 0.502 | 0.8680 | 0.7642 | 0.6593 | 3 | 3 | 3 |
|  | b | 0.447 | 0.441 | 0.667 | 0.502 | 0.504 | 0.511 | 0.1889 | 0.3342 | 0.2034 | 3 | 3 | 3 |
|  | c | 0.333 | 0.397 | 0.143 | 0.352 | 0.370 | 0.215 | 0.4926 | 0.7416 | 0.2328 | 3 | 3 | 3 |

* Not the same alleles. ** only AA and Aa genotypes
