# Supplementary material for: Genetic diversity and population structure of African village dogs based on microsatellite and immunity-related molecular markers
Source: PLoS One. 2018 Jun 25;13(6):e0199506. doi: 10.1371/journal.pone.0199506 (PMC6016929; doi:10.1371/journal.pone.0199506)
Supplement: S6 Table — (DOCX) [file pone.0199506.s011.docx]

| **FHC2010**  **CFA24** | **FHC2054**  **CFA12** | **FHC2079**  **CFA24** |
| --- | --- | --- |
| Allele MK MN LT  219 0.05000 0.00000 0.00000  223 0.00000 0.12903 0.14773  227 0.16000 0.31183 0.26136  231 0.41000 0.19355 0.19318  235 0.35000 0.36559 0.37500  239 0.00000 0.00000 0.02273  243 0.03000 0.00000 0.00000 | Allele MK MN LT  148 0.00000 0.00000 0.01136  151 0.08247 0.11111 0.03409  152 0.04124 0.03030 0.02273  155 0.13402 0.10101 0.14773  156 0.18557 0.04040 0.26136  159 0.04124 0.12121 0.00000  160 0.01031 0.01010 0.06818  163 0.10309 0.12121 0.03409  164 0.04124 0.04040 0.11364  167 0.12371 0.07071 0.05682  168 0.05155 0.03030 0.10227  171 0.04124 0.07071 0.04545  172 0.06186 0.03030 0.04545  175 0.04124 0.12121 0.02273  176 0.04124 0.10101 0.03409 | Allele MK MN LT  269 0.23000 0.22680 0.35165  273 0.26000 0.30928 0.12088  277 0.06000 0.22680 0.13187  281 0.13000 0.08247 0.10989  283 0.00000 0.00000 0.01099  285 0.04000 0.04124 0.03297  289 0.05000 0.03093 0.03297  293 0.21000 0.08247 0.20879  297 0.02000 0.00000 0.00000 |
| **PEZ1**  **CFA7** | **PEZ12**  **CFA3** | **PEZ20**  **unmapped** |
| Allele MK MN LT  106 0.15000 0.37500 0.26437  110 0.11000 0.06250 0.17241  114 0.46000 0.16667 0.18391  118 0.18000 0.23958 0.26437  122 0.10000 0.15625 0.11494 | Allele MK MN LT  260 0.04000 0.02062 0.05814  264 0.18000 0.25773 0.17442  268 0.19000 0.24742 0.22093  272 0.07000 0.32990 0.13953  276 0.15000 0.04124 0.05814  280 0.15000 0.03093 0.19767  284 0.14000 0.00000 0.01163  288 0.01000 0.00000 0.08140  292 0.03000 0.01031 0.03488  296 0.00000 0.04124 0.01163  300 0.02000 0.01031 0.00000  304 0.02000 0.01031 0.01163 | Allele MK MN LT  167 0.00000 0.04348 0.01266  171 0.14286 0.20652 0.26582  175 0.45918 0.25000 0.21519  179 0.11224 0.22826 0.29114  183 0.14286 0.19565 0.08861  187 0.02041 0.00000 0.01266  191 0.01020 0.01087 0.05063  194 0.04082 0.00000 0.00000  195 0.07143 0.06522 0.06329 |
| **PEZ3**  **CFA19** | **PEZ5**  **CFA12** | **PEZ6**  **CFA27** |
| Allele MK MN LT  114 0.00000 0.00000 0.01250  117 0.04040 0.18182 0.00000  120 0.06061 0.01010 0.12500  123 0.15152 0.16162 0.13750  126 0.08081 0.23232 0.32500  129 0.11111 0.04040 0.01250  132 0.06061 0.00000 0.07500  135 0.15152 0.15152 0.03750  138 0.03030 0.02020 0.15000  141 0.17172 0.14141 0.06250  150 0.01010 0.00000 0.00000  93 0.13131 0.06061 0.06250 | Allele MK MN LT  102 0.34021 0.55670 0.32584  106 0.25773 0.20619 0.21348  110 0.30928 0.15464 0.38202  114 0.03093 0.02062 0.01124  94 0.02062 0.06186 0.00000  98 0.04124 0.00000 0.06742 | Allele MK MN LT  168 0.03000 0.02105 0.03261  170 0.00000 0.01053 0.00000  172 0.11000 0.05263 0.05435  176 0.17000 0.21053 0.05435  180 0.11000 0.04211 0.27174  182 0.00000 0.01053 0.00000  184 0.24000 0.28421 0.23913  186 0.00000 0.11579 0.02174  188 0.29000 0.18947 0.21739  192 0.02000 0.01053 0.07609  196 0.02000 0.05263 0.02174  200 0.01000 0.00000 0.01087 |
| **PEZ8**  **CFA17** | **AHTk211**  **CFA26** | **CXX279**  **CFA22** |
| Allele MK MN LT  219 0.03061 0.04167 0.01136  223 0.17347 0.27083 0.20455  227 0.25510 0.11458 0.18182  231 0.13265 0.35417 0.17045  235 0.22449 0.15625 0.32955  239 0.16327 0.04167 0.10227  243 0.02041 0.02083 0.00000 | Allele MK MN LT  85 0.01000 0.00000 0.00000  87 0.26000 0.55000 0.43000  89 0.10000 0.05000 0.10000  91 0.47000 0.35000 0.38000  93 0.02000 0.01000 0.00000  95 0.14000 0.04000 0.09000 | Allele MK MN LT  114 0.03061 0.00000 0.00000  116 0.03061 0.13131 0.07000  118 0.41837 0.34343 0.35000  120 0.00000 0.01010 0.02000  122 0.07143 0.00000 0.03000  124 0.08163 0.03030 0.02000  126 0.24490 0.33333 0.37000  128 0.11224 0.14141 0.14000  132 0.01020 0.01010 0.00000 |
| **INU055**  **CFA10** | **REN169O18**  **CFA29** | **REN54P11**  **CFA18** |
| Allele MK MN LT  200 0.11111 0.01053 0.13542  208 0.05051 0.00000 0.00000  210 0.50505 0.69474 0.28125  212 0.06061 0.00000 0.00000  214 0.07071 0.02105 0.14583  216 0.01010 0.00000 0.00000  218 0.02020 0.00000 0.06250  220 0.15152 0.25263 0.33333  222 0.01010 0.02105 0.01042  224 0.00000 0.00000 0.02083  226 0.01010 0.00000 0.01042 | Allele MK MN LT  162 0.13000 0.15152 0.20000  164 0.17000 0.22222 0.20000  166 0.23000 0.34343 0.30000  168 0.34000 0.18182 0.15000  170 0.09000 0.04040 0.10000  172 0.04000 0.06061 0.05000 | Allele MK MN LT  222 0.04000 0.07216 0.13000  226 0.15000 0.06186 0.11000  228 0.07000 0.00000 0.02000  230 0.01000 0.00000 0.00000  232 0.02000 0.02062 0.01000  234 0.49000 0.51546 0.43000  236 0.09000 0.01031 0.16000  238 0.06000 0.13402 0.11000  240 0.01000 0.11340 0.02000  242 0.03000 0.04124 0.01000  244 0.02000 0.03093 0.00000  246 0.01000 0.00000 0.00000 |
| **AHT137**  **CFA11** | **AHTh260**  **CFA16** | **AHTk253**  **CFA23** |
| Allele MK MN LT  131 0.04082 0.16162 0.07000  133 0.08163 0.05051 0.01000  135 0.22449 0.16162 0.11000  137 0.12245 0.05051 0.01000  139 0.00000 0.02020 0.03000  143 0.00000 0.03030 0.03000  145 0.03061 0.02020 0.05000  147 0.15306 0.16162 0.42000  149 0.15306 0.24242 0.18000  151 0.12245 0.09091 0.02000  153 0.07143 0.01010 0.07000 | Allele MK MN LT  236 0.01000 0.00000 0.01000  238 0.01000 0.06000 0.05000  240 0.00000 0.09000 0.03000  242 0.21000 0.20000 0.30000  244 0.04000 0.19000 0.08000  246 0.46000 0.31000 0.33000  248 0.20000 0.14000 0.11000  250 0.00000 0.01000 0.06000  252 0.07000 0.00000 0.03000 | Allele MK MN LT  280 0.00000 0.01031 0.01000  282 0.05000 0.06186 0.03000  284 0.10000 0.01031 0.09000  286 0.25000 0.16495 0.22000  288 0.33000 0.35052 0.47000  290 0.14000 0.10309 0.09000  292 0.08000 0.26804 0.06000  294 0.01000 0.00000 0.00000  296 0.01000 0.01031 0.02000  298 0.03000 0.02062 0.01000 |
| **INRA21**  **CFA21** | **REN169D01**  **CFA14** | **AHT121**  **CFA13** |
| Allele MK MN LT  101 0.11000 0.14000 0.08000  103 0.00000 0.00000 0.03000  105 0.11000 0.02000 0.20000  107 0.01000 0.00000 0.00000  109 0.00000 0.01000 0.01000  115 0.01000 0.00000 0.00000  91 0.01000 0.00000 0.01000  95 0.32000 0.57000 0.36000  97 0.17000 0.19000 0.16000  99 0.26000 0.07000 0.15000 | Allele MK MN LT  202 0.19000 0.05000 0.02000  208 0.01000 0.02000 0.02000  210 0.02000 0.09000 0.05000  212 0.04000 0.06000 0.05000  214 0.00000 0.00000 0.03000  216 0.52000 0.68000 0.64000  218 0.02000 0.04000 0.02000  220 0.18000 0.06000 0.17000  222 0.02000 0.00000 0.00000 | Allele MK MN LT  100 0.10204 0.15152 0.14000  102 0.13265 0.09091 0.10000  104 0.10204 0.10101 0.11000  106 0.12245 0.03030 0.03000  108 0.01020 0.00000 0.02000  110 0.00000 0.00000 0.03000  112 0.01020 0.01010 0.05000  114 0.00000 0.00000 0.02000  86 0.00000 0.02020 0.00000  90 0.14286 0.07071 0.07000  92 0.03061 0.10101 0.01000  94 0.03061 0.04040 0.09000  96 0.07143 0.24242 0.13000  98 0.24490 0.14141 0.20000 |
| **AHTh171**  **CFA6** | **REN162C04**  **CFA7** | **REN247M23**  **CFA15** |
| Allele MK MN LT  214 0.02000 0.00000 0.00000  217 0.20000 0.10638 0.16327  219 0.14000 0.00000 0.07143  221 0.29000 0.29787 0.26531  223 0.11000 0.14894 0.20408  225 0.16000 0.14894 0.06122  227 0.02000 0.00000 0.02041  229 0.05000 0.03191 0.06122  231 0.00000 0.07447 0.01020  233 0.00000 0.19149 0.06122  237 0.01000 0.00000 0.08163 | Allele MK MN LT  192 0.01020 0.00000 0.02000  194 0.01020 0.01064 0.04000  196 0.01020 0.00000 0.01000  198 0.06122 0.01064 0.03000  200 0.04082 0.02128 0.04000  202 0.04082 0.08511 0.03000  204 0.38776 0.48936 0.35000  206 0.39796 0.30851 0.40000  208 0.02041 0.07447 0.05000  210 0.02041 0.00000 0.03000 | Allele MK MN LT  266 0.00000 0.01075 0.01000  268 0.55556 0.65591 0.56000  270 0.13131 0.13978 0.13000  272 0.16162 0.07527 0.20000  276 0.04040 0.00000 0.03000  278 0.11111 0.05376 0.03000  280 0.00000 0.05376 0.04000  282 0.00000 0.01075 0.00000 |
| **FHC2848**  **CFA2** | **INU005**  **CFA33** | **INU030**  **CFA12** |
| Allele MK MN LT  228 0.04000 0.00000 0.00000  230 0.06000 0.01053 0.16667  232 0.00000 0.00000 0.01042  234 0.01000 0.00000 0.00000  236 0.42000 0.24211 0.27083  238 0.23000 0.14737 0.12500  240 0.14000 0.42105 0.32292  242 0.03000 0.05263 0.04167  244 0.03000 0.00000 0.05208  246 0.03000 0.10526 0.01042  248 0.01000 0.02105 0.00000 | Allele MK MN LT  110 0.00000 0.00000 0.03000  112 0.06186 0.05000 0.06000  114 0.00000 0.01000 0.00000  122 0.00000 0.01000 0.00000  124 0.08247 0.08000 0.15000  126 0.40206 0.41000 0.41000  128 0.12371 0.08000 0.09000  130 0.15464 0.27000 0.12000  132 0.17526 0.09000 0.14000 | Allele MK MN LT  144 0.04082 0.10417 0.04000  146 0.27551 0.51042 0.25000  148 0.09184 0.05208 0.08000  150 0.45918 0.17708 0.47000  152 0.00000 0.03125 0.03000  154 0.12245 0.12500 0.13000  156 0.01020 0.00000 0.00000 |
