# Supplementary material for: Genetic diversity and population structure of African village dogs based on microsatellite and immunity-related molecular markers
Source: PLoS One. 2018 Jun 25;13(6):e0199506. doi: 10.1371/journal.pone.0199506 (PMC6016929; doi:10.1371/journal.pone.0199506)
Supplement: S7 Table — MK- Mt. Kulal. MN- Mt. Ngyiro. LK- Lake Turkana. (DOCX) [file pone.0199506.s012.docx]

| Locus | Chrom | Observed heterozygosity | | | Expected heterozygosity | | | P- value | | |
| --- | --- | --- | --- | --- | --- | --- | --- | --- | --- | --- |
|  |  | MK  (n= 50) | MN  (n= 50) | LT  (n= 50) | MK  (n= 50) | MN  (n= 50) | LT  (n= 50) | MK | MN | LT |
| FH2010 | CFA24 | 0.600 | 0.565 | 0.698 | 0.687 | 0.723 | 0.740 | 0.1976 | 0.0892 | 0.4486 |
| FH2054 | CFA12 | 0.854 | 0.714 | 0.750 | 0.907 | 0.916 | 0.880 | 0.0000 | 0.0000 | 0.0000 |
| FH2079 | CFA24 | 0.800 | 0.729 | 0.756 | 0.819 | 0.793 | 0.795 | 0.5098 | 0.1785 | 0.2551 |
| PEZ1 | CFA7 | 0.640 | 0.702 | 0.791 | 0.719 | 0.754 | 0.793 | 0.3158 | 0.3429 | 0.9767 |
| PEZ12 | CFA3 | 0.760 | 0.750 | 0.814 | 0.867 | 0.766 | 0.857 | 0.1704 | 0.0772 | 0.4213 |
| PEZ20 | unmapped | 0.633 | 0.761 | 0.737 | 0.736 | 0.807 | 0.794 | 0.1680 | 0.0047 | 0.5579 |
| PEZ3 | CFA19 | 0.878 | 0.755 | 0.769 | 0.887 | 0.847 | 0.833 | 0.3086 | 0.0681 | 0.0140 |
| PEZ5 | CFA12 | 0.667 | 0.542 | 0.682 | 0.727 | 0.626 | 0.706 | 0.3356 | 0.1831 | 0.3290 |
| PEZ6 | CFA27 | 0.600 | 0.717 | 0.804 | 0.812 | 0.826 | 0.817 | 0.0009 | 0.0976 | 0.9419 |
| PEZ8 | CFA17 | 0.796 | 0.660 | 0.744 | 0.817 | 0.768 | 0.786 | 0.3329 | 0.0155 | 0.9612 |
| AHTk211 | CFA26 | 0.480 | 0.540 | 0.660 | 0.688 | 0.577 | 0.659 | 0.0000 | 0.3166 | 0.6834 |
| CXX279 | CFA22 | 0.653 | 0.755 | 0.740 | 0.746 | 0.740 | 0.722 | 0.3834 | 0.4094 | 0.2880 |
| INU055 | CFA10 | 0.490 | 0.326 | 0.702 | 0.705 | 0.457 | 0.774 | 0.0000 | 0.0605 | 0.0162 |
| REN169O18 | CFA29 | 0.720 | 0.633 | 0.820 | 0.784 | 0.779 | 0.803 | 0.5376 | 0.0345 | 0.8196 |
| REN54P11 | CFA18 | 0.740 | 0.638 | 0.720 | 0.724 | 0.698 | 0.755 | 0.5562 | 0.0911 | 0.3094 |
| AHT137 | CFA11 | 0.898 | 0.837 | 0.800 | 0.867 | 0.856 | 0.772 | 0.1042 | 0.0363 | 0.9729 |
| AHTh260 | CFA16 | 0.680 | 0.740 | 0.840 | 0.705 | 0.804 | 0.782 | 0.6974 | 0.5170 | 0.4698 |
| AHTk253 | CFA23 | 0.860 | 0.660 | 0.680 | 0.797 | 0.771 | 0.717 | 0.0925 | 0.0126 | 0.0996 |
| INRA21 | CFA21 | 0.660 | 0.500 | 0.860 | 0.784 | 0.620 | 0.783 | 0.0104 | 0.0319 | 0.2949 |
| REN169DO1A | CFA14 | 0.540 | 0.480 | 0.480 | 0.665 | 0.523 | 0.560 | 0.0217 | 0.1780 | 0.0004 |
| AHT121 | CFA13 | 0.833 | 0.653 | 0.880 | 0.868 | 0.870 | 0.892 | 0.3340 | 0.0058 | 0.8236 |
| AHTh171 | CFA06 | 0.620 | 0.478 | 0.796 | 0.823 | 0.821 | 0.846 | 0.0000 | 0.0000 | 0.5123 |
| REN162CO4 | CFA07 | 0.612 | 0.574 | 0.720 | 0.690 | 0.659 | 0.716 | 0.4822 | 0.1933 | 0.9583 |
| REN247M23 | CFA15 | 0.490 | 0.478 | 0.620 | 0.640 | 0.544 | 0.632 | 0.0600 | 0.2644 | 0.7504 |
| FHC2848 | CFA02 | 0.740 | 0.617 | 0.708 | 0.751 | 0.736 | 0.782 | 0.0319 | 0.1290 | 0.3286 |
| INU005 | CFA33 | 0.688 | 0.520 | 0.800 | 0.766 | 0.743 | 0.771 | 0.3615 | 0.0015 | 0.1654 |
| INU030 | CFA12 | 0.571 | 0.660 | 0.700 | 0.695 | 0.685 | 0.698 | 0.0707 | 0.5727 | 0.3709 |

MK- Mt. Kulal. MN- Mt. Ngyiro. LK- Lake Turkana
