# Supplementary material for: Genetic diversity and population structure of African village dogs based on microsatellite and immunity-related molecular markers
Source: PLoS One. 2018 Jun 25;13(6):e0199506. doi: 10.1371/journal.pone.0199506 (PMC6016929; doi:10.1371/journal.pone.0199506)
Supplement: S8 Table — (DOCX) [file pone.0199506.s013.docx]

| Locus | Fis | Fit | Fst | Locus | Fis | Fit | Fst | Locus | Fis | Fit | Fst |
| --- | --- | --- | --- | --- | --- | --- | --- | --- | --- | --- | --- |
| FHC2010 | 0.12 | 0.15 | 0.03 | PEZ8 | 0.06 | 0.08 | 0.02 | INRA21 | 0.07 | 0.1 | 0.03 |
| FHC2054 | 0.13 | 0.15 | 0.02 | AHTk211 | 0.12 | 0.14 | 0.03 | REN169D01 | 0.13 | 0.15 | 0.02 |
| FHC2079 | 0.04 | 0.06 | 0.02 | CXX279 | 0.02 | 0.03 | 0.01 | AHT121 | 0.09 | 0.10 | 0.02 |
| PEZ1 | 0.05 | 0.09 | 0.04 | INU055 | 0.21 | 0.26 | 0.06 | AHTh171 | 0.23 | 0.25 | 0.02 |
| PEZ12 | 0.06 | 0.09 | 0.03 | REN169O18 | 0.07 | 0.09 | 0.01 | REN162C04 | 0.07 | 0.08 | 0.01 |
| PEZ20 | 0.08 | 0.10 | 0.03 | REN54P11 | 0.03 | 0.04 | 0.02 | REN247M23 | 0.12 | 0.13 | 0.01 |
| PEZ3 | 0.06 | 0.09 | 0.03 | AHT137 | -0.03 | 0.01 | 0.03 | FH2848 | 0.08 | 0.12 | 0.04 |
| PEZ5 | 0.07 | 0.10 | 0.03 | AHTh260 | 0.01 | 0.02 | 0.02 | INU005 | 0.11 | 0.13 | 0.01 |
| PEZ6 | 0.12 | 0.15 | 0.03 | AHTk253 | 0.03 | 0.05 | 0.02 | INU030 | 0.07 | 0.11 | 0.04 |
|  | Fis | | | | Fit | | | Fst | | | |
| Mean | 0.081 | | | | 0.105 | | | 0.026 | | | |
| SEM | 0.011 | | | | 0.011 | | | 0.002 | | | |
