# Supplementary material for: Genetic diversity and population structure of African village dogs based on microsatellite and immunity-related molecular markers
Source: PLoS One. 2018 Jun 25;13(6):e0199506. doi: 10.1371/journal.pone.0199506 (PMC6016929; doi:10.1371/journal.pone.0199506)
Supplement: S9 Table — (DOCX) [file pone.0199506.s014.docx]

| **DRB**  **CFA12** | **DQA**  **CFA12** | **DQB**  **CFA12** |
| --- | --- | --- |
| Allele MK MN LT  00101 0.02174 0.04255 0.13542  00201 0.03261 0.0000 0.02083  00301 0.00000 0.00000 0.02083  00401 0.06522 0.02128 0.08333  00601 0.00000 0.12766 0.02083  00802 0.02174 0.10638 0.00000  00901 0.22826 0.13830 0.16667  01101 0.00000 0.02128 0.00000  01201 0.03261 0.00000 0.00000  01301 0.00000 0.10638 0.00000  01501 0.08696 0.17021 0.27083  01801 0.06522 0.01064 0.01042  02001 0.02174 0.12766 0.03125  04001 0.01087 0.00000 0.00000  04801 0.03261 0.00000 0.00000  07401 0.00000 0.01064 0.00000  07901 0.00000 0.00000 0.01042  092v 0.10870 0.01064 0.04167  098v 0.00000 0.00000 0.01042  10801 0.23913 0.04255 0.07292  108v 0.01087 0.00000 0.04167  new 0.01087 0.00000 0.00000  ph211 0.00000 0.04255 0.00000  ph271 0.01087 0.02128 0.02083  ph486 0.00000 0.00000 0.04167 | Allele MK MN LT  00101 0.56667 0.30851 0.42708  00201 0.04444 0.07447 0.10417  002v 0.01111 0.00000 0.00000  00301 0.02222 0.10638 0.04167  00401 0.04444 0.12766 0.03125  00402 0.03333 0.04255 0.01042  005011 0.00000 0.13830 0.02083  00601 0.23333 0.20213 0.34375  00901 0.03333 0.00000 0.02083  01001 0.01111 0.00000 0.00000 | Allele MK MN LT  00101 0.04839 0.00000 0.02128  00201 0.14516 0.15957 0.18085  00701 0.00000 0.13830 0.02128  008011 0.16129 0.01064 0.05319  008012 0.22581 0.04255 0.11702  00802 0.19355 0.10638 0.12766  01302 0.00000 0.02128 0.00000  01303 0.00000 0.12766 0.03191  01305 0.00000 0.03191 0.02128  01501 0.08065 0.02128 0.08511  02301 0.04839 0.08511 0.10638  03101 0.01613 0.02128 0.08511  03801 0.00000 0.00000 0.04255  05401 0.03226 0.05319 0.02128  05701 0.03226 0.07447 0.08511  ug115 0.01613 0.10638 0.00000 |
