# Supplementary material for: Genetic diversity and population structure of African village dogs based on microsatellite and immunity-related molecular markers
Source: PLoS One. 2018 Jun 25;13(6):e0199506. doi: 10.1371/journal.pone.0199506 (PMC6016929; doi:10.1371/journal.pone.0199506)
Supplement: S10 Table — (DOCX) [file pone.0199506.s015.docx]

|  | Genes | Number of alleles | Observed heterozygosity | Expected heterozygosity | P- value |
| --- | --- | --- | --- | --- | --- |
| Mt.Kulal  (n=41) | DRB1 | 16 | 0.783 | 0.867 | 0.2058 |
|  | DQA1 | 9 | 0.600 | 0.624 | 0.0872 |
|  | DQB1 | 11 | 0.871 | 0.865 | 0.2335 |
| Mt.Ngyiro  (n=47) | DRB1 | 15 | 0.894 | 0.899 | 0.2037 |
|  | DQA1 | 7 | 0.830 | 0.819 | 0.5543 |
|  | DQB1 | 14 | 0.894 | 0.906 | 0.3630 |
| Lake Turkana  (n=47) | DRB1 | 16 | 0.938 | 0.869 | 0.7247 |
|  | DQA1 | 8 | 0.688 | 0.692 | 0.7522 |
|  | DQB1 | 14 | 0.936 | 0.906 | 0.5817 |
