# Supplementary material for: Genetic diversity and population structure of African village dogs based on microsatellite and immunity-related molecular markers
Source: PLoS One. 2018 Jun 25;13(6):e0199506. doi: 10.1371/journal.pone.0199506 (PMC6016929; doi:10.1371/journal.pone.0199506)
Supplement: S11 Table — MK- Mt. Kulal. MN- Mt. Ngyiro. LK- Lake Turkana* Not the same alleles. ** only AA and Aa genotypes. (DOCX) [file pone.0199506.s016.docx]

|  |  | Observed heterozygosity | | | Expected heterozygosity | | | P- value | | | Number of genotypes | | | MAF | | |
| --- | --- | --- | --- | --- | --- | --- | --- | --- | --- | --- | --- | --- | --- | --- | --- | --- |
|  |  |  |  |  |  |  |  |  | | |  | | |  |  |  |
|  |  | MK  (n=50) | MN  (n=50) | LT  (n=50) | MK  (n=50) | MN  (n=50) | LT  (n=50) | MK | MN | LT | MK  (n=50) | MN  (n=50) | LT  (n=50) | MK  (n=50) | MN  (n=50) | LT  (n=50) |
| *NOS3* | HpyCH4V | 0.060 | 0.220 | 0.040 | 0.059 | 0.228 | 0.040 | 1.0000 | 1.0000 | 1.0000 | 2** | 3 | 2** | 0.030 | 0.130 | 0.020 |
|  | BsaJI | 0.400 | 0.380 | 0.320 | 0.347 | 0.460 | 0.368 | 0.4136 | 0.2312 | 0.4371 | 3 | 3 | 3 | 0.220 | 0.350 | 0.240 |
| *IL6* | BseYI | 0.061 | 0.163 | 0.020 | 0.060 | 0.151 | 0.020 | 1.0000 | 1.0000 | 1.0000 | 2** | 2** | 2** | 0.031 | 0.082 | 0.010 |
|  | HpaI | 0.260 | 0.245 | 0.060 | 0.285 | 0.247 | 0.058 | 0.6108 | 1.0000 | 1.0000 | 3 | 3 | 2** | 0.170 | 0.143 | 0.030 |
| *TLR1* | Sau96I | 0.140 | 0.490 | 0.388 | 0.198 | 0.480 | 0.421 | 0.0859 | 1.0000 | 0.7306 | 3 | 3 | 3 | 0.110 | 0.388 | 0.296 |
| *TLR2* | NspI | 0.060 | 0.140 | 0.040 | 0.059 | 0.132 | 0.040 | 1.0000 | 1.0000 | 1.0000 | 2** | 2** | 2** | 0.030 | 0.070 | 0.020 |
| *TLR4* | XcmI | 0.500 | 0.340 | 0.560 | 0.447 | 0.505 | 0.502 | 0.5226 | 0.0265 | 0.5684 | 3 | 3 | 3 | 0.330 | 0.490* | 0.460 |
|  | HinPI | 0.540 | 0.320 | 0.440 | 0.495 | 0.407 | 0.502 | 0.5726 | 0.1608 | 0.4099 | 3 | 3 | 3 | 0.430* | 0.280 | 0.460 |
| *TLR7* | BtsCI | 0.180 | 0.102 | 0.120 | 0.495 | 0.498 | 0.476 | 0.0000 | 0.0000 | 0.0000 | 3 | 3 | 3 | 0.430 | 0.439* | 0.380 |
| *TLR9* | NgoMIV | 0.260 | 0.220 | 0.340 | 0.228 | 0.198 | 0.416 | 0.5803 | 1.0000 | 0.3010 | 3 | 2** | 3 | 0.130 | 0.110 | 0.290 |
|  | BseYI | 0.440 | 0.360 | 0.380 | 0.440 | 0.453 | 0.379 | 1.0000 | 0.2073 | 1.0000 | 3 | 3 | 3 | 0.320 | 0.340 | 0.250 |
| *LY96* | BtsCI | 0.220 | 0.563 | 0.319 | 0.258 | 0.468 | 0.395 | 0.2840 | 0.2144 | 0.2577 | 3 | 3 | 3 | 0.150 | 0.365 | 0.266 |
|  | Hpy166II | 0.380 | 0.571 | 0.540 | 0.416 | 0.495 | 0.495 | 0.7268 | 0.3869 | 0.5724 | 3 | 3 | 3 | 0.290 | 0.429* | 0.430 |
| *MYD88* | BglI | 0.440 | 0.500 | 0.540 | 0.505 | 0.503 | 0.503 | 0.4091 | 1.0000 | 0.7770 | 3 | 3 | 3 | 0.500 | 0.470* | 0.470 |
|  | BamHI | 0.520 | 0.320 | 0.500 | 0.504 | 0.505 | 0.505 | 1.0000 | 0.0103 | 1.0000 | 3 | 3 | 3 | 0.480* | 0.500 | 0.490 |
|  | ApaLI | 0.320 | 0.320 | 0.360 | 0.347 | 0.323 | 0.389 | 0.6814 | 1.0000 | 0.7157 | 3 | 3 | 3 | 0.220 | 0.200 | 0.260 |

MK- Mt. Kulal. MN- Mt. Ngyiro. LK- Lake Turkana

* Not the same alleles. ** only AA and Aa genotypes
