# Supplementary material for: Genetic diversity and population structure of African village dogs based on microsatellite and immunity-related molecular markers
Source: PLoS One. 2018 Jun 25;13(6):e0199506. doi: 10.1371/journal.pone.0199506 (PMC6016929; doi:10.1371/journal.pone.0199506)
Supplement: S12 Table — NS Non-significant, **P<0.01. (DOCX) [file pone.0199506.s017.docx]

| Reynolds distance | Msat | MHC | SNP | Spearman's correlation coefficient |
| --- | --- | --- | --- | --- |
| Kulal x Ngyiro | 0.045 | 0.065 | 0.062 | Msat x MHC |
| Ngyiro x Turkana | 0.04 | 0.037 | 0.668 | 0.943** |
| Kulal x Turkana | 0.031 | 0.036 | 0.746 | Msat x SNP |
| Kulal x Survivor | 0.011 | 0.014 | 0.425 | -0.600^NS^ |
| Ngyiro x Survivor | 0.055 | 0.078 | 0.351 | MHC x SNP |
| Turkana x Survivor | 0.035 | 0.046 | 0.725 | -0.543^NS^ |

^NS^ Non-significant, **P<0.01
