# Supplementary material for: Genetic diversity and population structure of African village dogs based on microsatellite and immunity-related molecular markers
Source: PLoS One. 2018 Jun 25;13(6):e0199506. doi: 10.1371/journal.pone.0199506 (PMC6016929; doi:10.1371/journal.pone.0199506)
Supplement: S13 Table — observed (Ho) and expected (He) heterozygosities and loci out of HWE. (DOCX) [file pone.0199506.s018.docx]

|  | Mean number alleles | Observed heterozygosity | Expected heterozygosity | Number of loci out of HWE | Reference |
| --- | --- | --- | --- | --- | --- |
| Kenya dog | 9.5 | 0.686 | 0.769 | 15 | This study |
| (n=150) |  |  |  |  |  |
| Mt.Kulal (n=50)/ | 8.3/ | 0.685/ | 0.766/ | 8/ | This study |
| Mt. Ngyiro (n=50)/ | 7.6/ | 0.629/ | 0.730/ | 10/ |  |
| Lake Turkana (n=50) | 8.2 | 0.743 | 0.765 | 4 |  |
| Uganda/ |  |  | 0.660/ |  | Boyko et al. |
| Namibia |  |  | 0.63 |  | 2009 |
| (n= 30/60) |  |  |  |  |  |
| Kintamani/ | 7.0/ | 0.681/ | 0.700/ |  | Puja et al. |
| Bali street dogs | 7.7 | 0.692 | 0.746 |  | 2005 |
| (n=40/40) |  |  |  |  |  |
| Australian dingo |  | 0.426 | 0.511 | 12 | Irion et al. |
|  |  |  |  |  | 2005 |
| The Korean native aboriginal dogs/ | 5.5/ | 0.717/ | 0.718/ | 3 | Kim et al. 2001 |
| Sakhalin/ |  |  |  |  |  |
| Taiwanese native dogs | 5.25/ | 0.558/ | 0.637/ | 2 |  |
| (n= 15/15/16) | 4 | 0.539 | 0.624 | 3 |  |
| South- African bred German shepherd dogs | 5.4 | 0.585 | 0.61 |  | Coutts & Harley 2009 |
| Outbred dogs | 9.9 | 0.748 | 0.831 |  |  |
| (n=73/156) |  |  |  |  |  |
|  |  |  |  |  |  |
