# Supplementary material for: Genetic diversity and population structure of African village dogs based on microsatellite and immunity-related molecular markers
Source: PLoS One. 2018 Jun 25;13(6):e0199506. doi: 10.1371/journal.pone.0199506 (PMC6016929; doi:10.1371/journal.pone.0199506)
Supplement: S14 Table — 1* monomorphic locus. (DOCX) [file pone.0199506.s019.docx]

|  | Kenya dogs | European dogs | Basset | Bernese Mountain Dog | European Terrier | Caucasian Shepherd  Dog |
| --- | --- | --- | --- | --- | --- | --- |
|  | (n=150) | (n=68) | (n=29) | (n= 51) | (n=14) | (n= 27) |
| Observed  heterozygosity | 0.721 | 0.629 | 0.593 | 0.486 | 0.514 | 0.741 |
| Expected  heterozygosity | 0.811 | 0.768 | 0.604 | 0.553 | 0.473 | 0.715 |
| Number of observed alleles | 9.4 | 8.5 | 3.8 | 5.1 | 2.6 | 5.5 |
| Number of loci out of HWE | 6 | 7 | 2 | 3 | 1* | 2 |

1* monomorphic locus
