# Supplementary material for: Genetic diversity and population structure of African village dogs based on microsatellite and immunity-related molecular markers
Source: PLoS One. 2018 Jun 25;13(6):e0199506. doi: 10.1371/journal.pone.0199506 (PMC6016929; doi:10.1371/journal.pone.0199506)
Supplement: S15 Table — (DOCX) [file pone.0199506.s020.docx]

| SNP marker | Tajima´s D | p |
| --- | --- | --- |
| *MYD88/a* | 1.746 | Non-significant |
| *MYD88/b* | 1.855 | Non-significant |
| *MYD88/c* | 0.807 | Non-significant |
| *LY96/a* | 0.843 | Non-significant |
| *LY96/b* | 1.441 | Non-significant |
| *TLR1* | 0.973 | Non-significant |
| *TLR4/a* | 1.834 | Non-significant |
| *NOS3b* | -0.045 | Non-significant |
| *DQA* | -0.152 | Non-significant |
| *DQB* | -0.721 | Non-significant |
